# Aboral cell types of *Clytia* and coral larvae have shared features and link taurine to the regulation of settlement

**DOI:** 10.1126/sciadv.adv1159

**Published:** 2025-05-16

**Authors:** Julia Ramon-Mateu, Anna Ferraioli, Núria Teixidó, Isabelle Domart-Coulon, Evelyn Houliston, Richard R. Copley

**Affiliations:** ^1^ Laboratoire de Biologie du Développement de Villefranche-sur-mer (LBDV), Sorbonne Université, CNRS, 06230 Villefranche-sur-mer, France.; ^2^ National Institute of Marine Biology, Ecology and Biotechnology, Ischia Marine Center, Stazione Zoologica Anton Dohrn, Ischia, Naples, Italy.; ^3^ Laboratoire d’Océanographie de Villefranche (LOV), Sorbonne Université, CNRS, 06230 Villefranche-sur-mer, France.; ^4^ Laboratoire Molécules de Communication et Adaptation des Microorganismes (MCAM) (UMR7245), Muséum National d’Histoire Naturelle (MNHN), CNRS, CP54, 63 Rue Buffon, 75005 Paris, France.

## Abstract

Larval settlement is of interest both for ecologists and for evolutionary biologists, who have proposed that anterior sensory systems for substrate selection provided the basis for animal brains. Nevertheless, the cellular and molecular regulation of settlement, including in Cnidaria (corals, jellyfish, sea anemones, and hydroids), is not well understood. We generated and compared anterior (aboral) transcriptomes and single-cell RNA sequencing datasets from the planula larvae of three cnidarian species: the jellyfish *Clytia hemisphaerica* and the corals *Astroides calycularis* and *Pocillopora acuta*. Integrating these datasets and characterizing aboral cell types, we defined common cellular features of the planula aboral end and identified clade-specific specializations in cell types. Among shared features were genes implicated in taurine uptake and catabolism expressed in distinct specialized aboral cell types. In functional assays using both *Clytia* and *Astroides* planulae, exogenous taurine inhibited settlement. These findings define the molecular and cellular architecture of the planula aboral pole and implicate localized taurine destruction in regulating settlement.

## Teaser

Larvae of jellyfish and corals have specialized sensory cells with shared features potentially linked to settlement regulation.

## INTRODUCTION

**Fig. 1.**
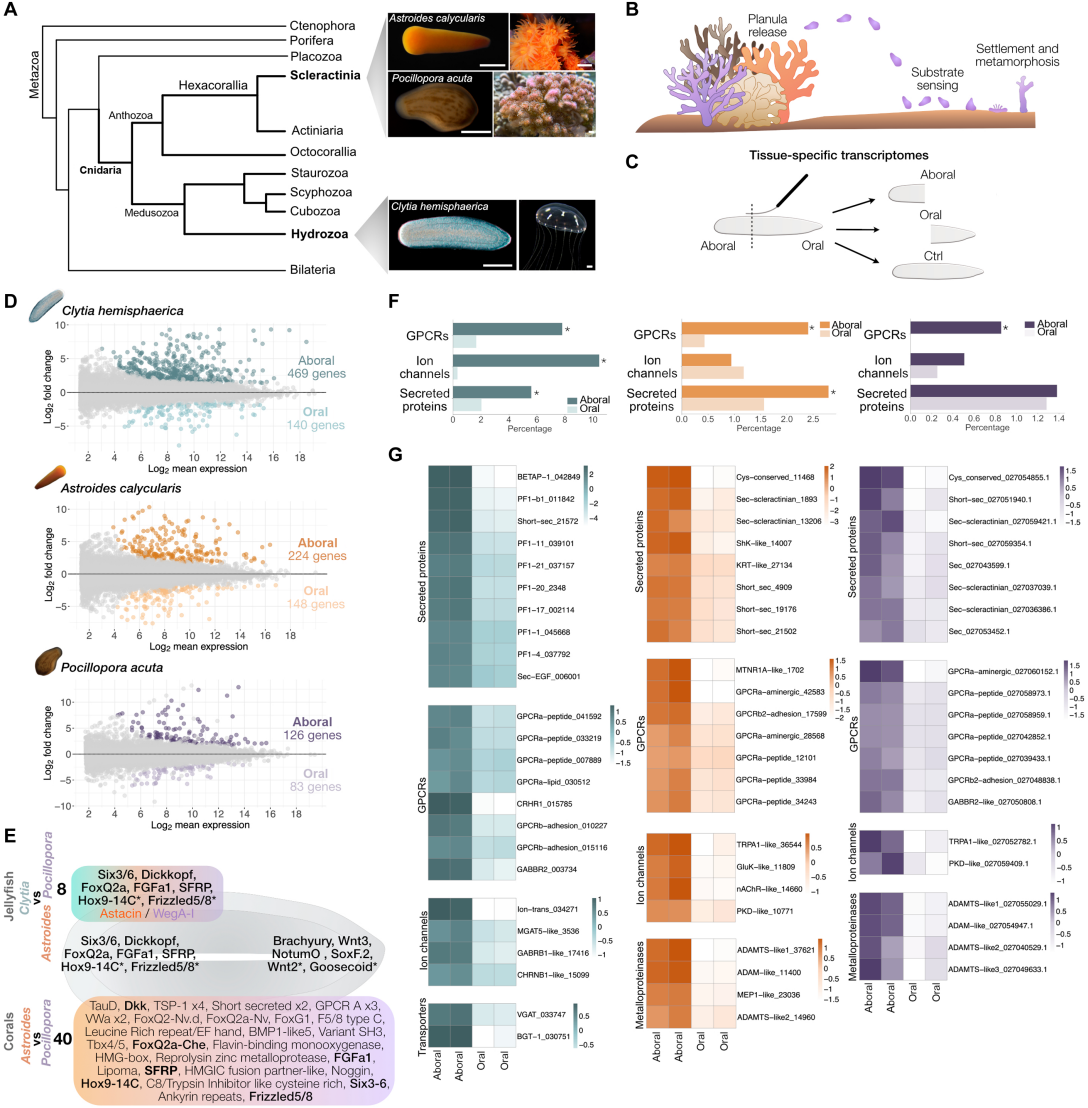
Planula aboral transcriptomes of a hydrozoan and two anthozoan species. (**A**) Phylogenetic placement of our cnidarian species of study. Topology adapted from (*111*, *112*). Images of *Clytia* reprinted from (*113*) with permission from Elsevier. *Astroides* adult colony image credit: C. Carbonne. Scale bars, 500 μm for *Astroides* and *Pocillopora* planulae; 100 μm for *Clytia* planula; and 1 mm for *Astroides*, *Pocillopora*, and *Clytia* adult stages. (**B**) Schematic of the phases of the settlement response in cnidarians. (**C**) Schematic of planula bisection. (**D**) MA plot showing differential gene expression between aboral and oral ends in each species. Each point represents a gene, with the *x* axis indicating average expression and the *y* axis showing log fold change. Significantly differentially expressed genes are highlighted in green (*Clytia*), orange (*Astroides*), and purple (*Pocillopora*). Adjusted *P* value (*P*_adj_) < 0.05; fold change (FC) > 1. *P* values were adjusted using the Benjamini-Hochberg method (DESeq2 default). (**E**) Schematic of a planula larva showing shared aborally and orally enriched genes from the differential gene expression analysis. The top panel highlights genes shared between *Clytia* and the two corals, and the bottom panel shows those shared between the two corals (*P*_adj_ < 0.05, FC > 1). Asterisks indicate fold change > 0 for one or more species. (**F**) Percentage of genes of selected types present in the aborally/orally enriched gene sets. Asterisks indicate significant differences between the aboral and oral sets (*P* < 0.05, Fisher’s exact test). (**G**) Heatmaps showing gene expression level (normalized counts) in the aboral/oral RNA-seq data for selected genes (see full aborally enriched datasets in table S1). Each condition (aboral/oral) includes two biological replicates. Gene names are based on orthology assignments or Pfam domains when orthology is unclear. Trailing numbers are unique gene identifiers from this project.

Settlement is a key event in the life cycle of cnidarians (jellyfish, hydroids, corals, sea anemones, etc.; Fig. 1, A and B) and other marine species, pivotal in the transition between a pelagic dispersive larval stage and a benthic adult stage. Cnidarian planula larvae from both of the main clades, Medusozoa and Anthozoa (Fig. 1A), are morphologically simple, consisting of outer ectoderm and inner gastroderm layers in the form of a tapered cylinder. Cells located at the anterior (aboral) end, the front relative to the swimming direction, are likely to mediate settlement. Aboral but not oral fragments of planulae from the hydroid *Hydractinia* (Medusozoa and Hydrozoa) are able to respond to environmental cues and start metamorphosis (*1*). Histological and ultrastructural analyses have described cells at the aboral pole of many cnidarian planulae containing secretory vesicles, implicated in substrate adhesion (*2*). An aboral concentration of putative sensory cells and an apical nerve plexus have also been described in various species (*3*–*5*), suggesting that sensory activity and integration of sensory information occur in the aboral region. Planulae of some anthozoan species, including the model *Nematostella vectensis*, bear a presumed sensory organ consisting of apical tuft cells carrying long specialized cilia, surrounded by flask-shaped neural cells (*6*, *7*). In hydrozoan planulae, the broad, aboral end of the larva is enriched with putative neurosensory cells producing GLWamide neuropeptides, known to be active in stimulating settlement and the accompanying marked cell reorganizations of metamorphosis (*3*, *8*–*11*).

A more detailed understanding of the cellular and molecular composition of the planula aboral end would likely yield insights into the settlement response. As well as enabling inquiry into the origins of animal nervous systems, which may have arisen to coordinate this process (*12*, *13*), such knowledge could be valuable in the context of ecosystem management for coral reefs. In this study, we generated and compared bulk transcriptomes from the aboral and oral ends of bisected planula larvae and single-cell RNA sequencing (scRNA-seq) data of the same larval stages. We used three species from Medusozoa—the hydrozoan jellyfish *Clytia hemisphaerica*—and Anthozoa—the scleractinian brooding stony corals *Astroides calycularis* and *Pocillopora acuta* (Fig. 1A). *Clytia* is a laboratory model species with larvae available from daily spawnings (*14*). *Astroides* is an endemic Mediterranean coral for which larvae were obtained from the natural annual release produced by wild populations, and *Pocillopora* is an Indo-Pacific, aquarium-propagated tropical coral that releases larvae monthly (*15*, *16*). We identified and characterized aboral cell types of each species, with a focus on neural and neurosecretory cells. From cross-species comparisons, we identified a conserved aborally expressed gene likely to be involved in taurine catabolism, and we demonstrate that taurine itself inhibits larval settlement. Together, our results allow us to propose a possible mechanism by which taurine mediates settlement in cnidarian larvae.

## RESULTS

### Aborally enriched gene sets from the planula of three cnidarian species

We manually bisected planula larvae of *Clytia*, *Astroides*, and *Pocillopora* and generated bulk RNA-seq data from pooled separated halves (Fig. 1C; see the Materials and Methods for details). Principal component analysis (PCA) of the most variably expressed genes showed, in all cases, that aboral and oral transcriptomes were distinct (fig. S1). Differential gene expression analysis between the aboral and oral ends of each species detected 469, 224, and 126 genes significantly enriched in the aboral transcriptomes of *Clytia*, *Astroides*, and *Pocillopora*, respectively (Fig. 1D; gene lists provided in table S1). The lower number of aborally enriched genes detected from the coral transcriptome data compared to *Clytia* is likely to be due to a combination of biological and technical differences. These members of distantly related cnidarian groups show differences in planula architecture: The planulae of the two scleractinian coral species are substantially larger than those of *Clytia* larvae (both more than 1 mm in length versus 200 to 300 μm) and show greater variability in shape, so specific aboral pole cell types would be more diluted within the aboral “half” fragments. The pooling of larvae from multiple coral mother colonies—likely with different genotypes—and from slightly different developmental stages compared to the synchronously developing and genotypically homogeneous *Clytia* larvae may have also contributed to this difference. For all species, fewer genes (140, 148, and 83) were detected as preferentially expressed in the oral halves.

### Shared features of aboral transcriptomes across species

Analysis of one-to-one orthologs (see the Materials and Methods) in common identified a small set of genes that were aborally enriched across all three species (Fig. 1E). These are all known anteriorly/apically/aborally expressed genes coding for Wnt and fibroblast growth factor (FGF) signaling pathway components or transcription factors responsible for patterning the anterior region in cnidarians, protostomes, and deuterostomes: the transcription factors Six3/6, FoxQ2A (*17*–*23*), and Hox9-14C (*18*, *24*–*27*); the FGFa1 ligand (*28*, *29*); a Frizzled 5/8 receptor (*30*, *31*); and the small secreted antagonists of the Wnt pathway SFRP (*18*, *27*, *30*, *32*–*35*) and Dickkopf (*27*, *36*). These results demonstrate a conserved oral-aboral patterning system across diverse cnidarian planulae and confirm that our cutting data are able to identify known aboral markers.

In addition to patterning genes conserved in all three species, we found recurring enriched aboral expression of genes coding for members of other types of protein families. We found genes with potential roles in sensing and signaling as common features of the aboral pole, including a variety of small secreted proteins, G protein–coupled receptors (GPCRs) and ligand-gated ion channels (Fig. 1F).

Among the highest aborally enriched genes in the *Clytia* planula were several homologous genes coding a family of short secreted proteins, which we term PF1 (Fig. 1G and fig. S2). We were unable to detect significant sequence similarity to non-*Clytia* proteins, and structure prediction with AlphaFold2 followed by database searching with FoldSeek (*37*–*39*) did not reveal similarity to existing protein three-dimensional (3D) structures (fig. S2C). Analysis of PF1 expression across the *Clytia* life cycle using earlier bulk transcriptome data (*40*) showed that their expression is largely restricted to the planula stage. Different secreted proteins, apparently unique to Anthozoa, were similarly present in the planula aboral transcriptomes of the two corals (fig. S2, B and D).

GPCRs were enriched in the aboral gene sets relative to the oral gene sets in all three species (Fig. 1F). Comparison of the aboral GPCR sets between the three species identified shared receptors between the two coral species but did not find receptors shared between *Clytia* and the corals, either because of the absence of direct one-to-one orthologs or because, when present, these orthologs were not enriched in the aboral region. However, a few aborally enriched *Clytia* receptors did show a corresponding aborally enriched ortholog in one of the coral species. Notably, both *Clytia* and *Pocillopora* included a metabotropic-like class C GPCR related (although not as a one-to-one ortholog) to γ-aminobutyric acid type B receptors (GABA_B_Rs) (fig. S3). In addition, in *Clytia*, we identified a neurotransmitter-gated ion channel with similarity to GABA_A_Rs, one amino acid transporter similar to GABA/glycine vesicular amino acid transporters (vGATs), and one sodium:neurotransmitter symporter channel similar to the sodium- and chloride-dependent betaine/GABA transporter (BGT-1) (Fig. 1G). Sustained activation of GABA_B_R signaling has been found to arrest metamorphosis of the *Nematostella* planula (*41*). This suggests a conserved role of GABA (or a related molecule) in the planula nervous system, potentially involved in the regulation of the settlement response in both *Clytia* and the corals.

We noted that all three aboral datasets included genes encoding the Pfam TauD domain (“Taurine catabolism dioxygenase domain”), a subfamily of a broader class of 2-oxoglutarate–dependent oxygenases (*42*). The TauD domain is found in enzymes involved in the catabolism of taurine, an amino sulfonic acid involved in physiological functions, including cell volume regulation, neuroprotection, and neurotransmission (*43*–*45*), but also in other proteins. Genes encoding this domain are expressed in the apical organ of *Nematostella* (*30*) and the apical domain of the planula of two *Acropora* species (*27*). Phylogenetic analysis indicates that all are closely related (fig. S4).

### Aboral cells in the *Clytia* planula are specialized neural and neurosecretory types

**Fig. 2.**
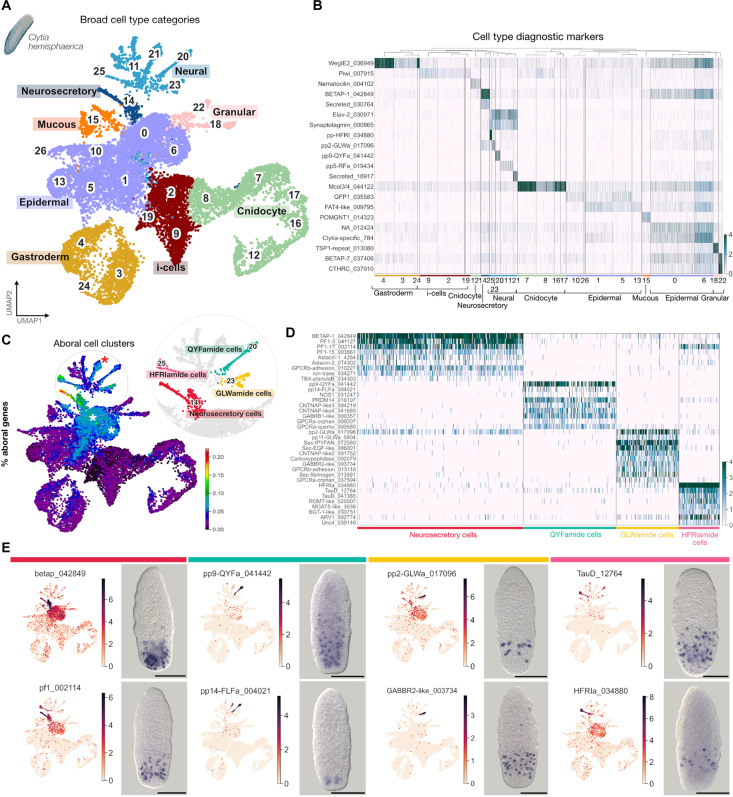
Aborally enriched cells in the *Clytia* planula. (**A**) Two-dimensional UMAP representation of *Clytia* planula scRNA-seq data with cell clusters color coded according to eight broad cell type classes: epidermal, gastrodermal, neural, neurosecretory, interstitial cells (i-cells), mucous, granular, and cnidocyte (developing and mature). Codes for class colors are retained in (B). (**B**) Heatmap of diagnostic marker genes for each cell type. Gene identifiers are plotted on the *y* axis, and cells are plotted on the *x* axis grouped by the annotation. Gene names are based on orthology assignments or Pfam domains when orthology is unclear. Trailing numbers are unique gene identifiers from this project. See full cluster marker gene lists in table S2. (**C**) UMAP plot of the *Clytia* planula cells, with cells colored on the basis of the percentage of expression of aborally enriched genes. Warmer colors indicate a higher proportion of aboral gene counts relative to total gene counts per cell. Aborally enriched gene cell clusters are highlighted in different colors in the upper right inset. The red asterisk indicates the “Nkd2d neural” cluster, and white asterisks indicate the two epidermal clusters. *P* values for aboral enrichment significance (Fisher’s exact test) are as follows: neurosecretory cells, 2.47 × 10^−72^; HFRIamide cells, 8.48 × 10^−23^; GLWamide cells, 4.12 × 10^−20^; QYFamide cells, 9.62 × 10^−10^; Nk2d cells, 8.47 × 10^−13^; epidermal cluster 0, 3.04 × 10^−10^; epidermal cluster 6, 2.41 × 10^−15^. (**D**) Heatmap of marker genes for each aboral cell cluster (complete gene list in table S3). Cell type annotation labels and colors correspond to (C). (**E**) UMAP plots highlighting log-normalized expression levels in each cell of selected aboral cell cluster markers and corresponding ISH patterns. Scale bars, 100 μm.

For scRNA-seq, we dissociated and fixed cells from *Clytia* planulae, as previously described (*46*) at two time points, 52 hours postfertilization (hpf; late 2-day planulae) and 66 hpf (early 3-day planulae), and processed them using the 10x Genomics platform. We generated four libraries that were merged and integrated with our existing scRNA-seq dataset of the *Clytia* planula larva (*46*) using Harmony (*47*). After filtering steps (see the Materials and Methods), the final dataset represented 14,627 cells expressing a total of 17,835 genes. Leiden clustering (*48*) generated 27 distinct clusters (fig. S5, A and B). These can be grouped into eight broad cell type classes: epidermal, gastrodermal, neural, neurosecretory, interstitial cells [“i-cells,” which are hydrozoan multipotent stem cells (*49*, *50*)], mucous, granular, and cnidocytes (cnidarian stinging cells, developing and mature) (Fig. 2, A and B). These are described in more detail elsewhere (*46*). All 27 clusters include cells from the different libraries including the two planula stages (fig. S5C).

To identify aboral cell types, we examined the strength of association between aborally enriched genes (table S1) and our cell clusters (table S2). Seven cell clusters showed an enrichment in aboral genes (Fig. 2C). Four of these showed a neural signature defined by expression of the pan-neural marker ELAV and other neural-related genes including synaptotagmin-like genes, neuropeptide precursors, and ion channels (cluster 20, QYFamide cells; cluster 21, Nk2d cells; cluster 23, GLWamide cells; cluster 25, HFRIamide cells). Cells in the Nk2d cluster appear to be heterogeneous (fig. S6). Individual marker genes for this cluster showed one of two profiles in both the aboral/oral transcriptome data (either aborally enriched or uniform) and the single-cell atlas [enriched in cells on left versus right sides of the cluster’s uniform manifold approximation and projection (UMAP) projection]. Cells of this cluster may thus be widely distributed but include an aboral subpopulation expressing the Nk2d transcription factor, a short secreted protein, two class A GPCRs, and two ion transporters. A fifth cluster was assigned as neurosecretory (cluster 14); it also expressed genes associated with neural function including synaptotagmin-like and calcium signaling–related genes, but also many secreted proteins.

In addition to these neural/neurosecretory aboral cell clusters, two clusters with epidermal-type signatures (clusters 0 and 6) showed high expression of aborally enriched genes. A few genes, notably ones coding for short secreted peptides, showed very high expression in these clusters, while others were expressed across many clusters (fig. S7A). In situ hybridization (ISH) for some of these genes revealed expression throughout the aboral epidermis, with a more intense signal in a band positioned about two thirds along the oral-aboral axis (fig. S7B). These clusters may thus represent specialized epidermal cells positioned at this location. However, the marker gene lists for clusters 0 and 6 also include genes highly expressed in other clusters, so we cannot rule out that they contain cell barcodes associated with ambient mRNA during sample preparation.

### Specializations of the aboral neural and neurosecretory cells

We further investigated the distinct character of each of our *Clytia* aboral cell types.

**Fig. 3.**
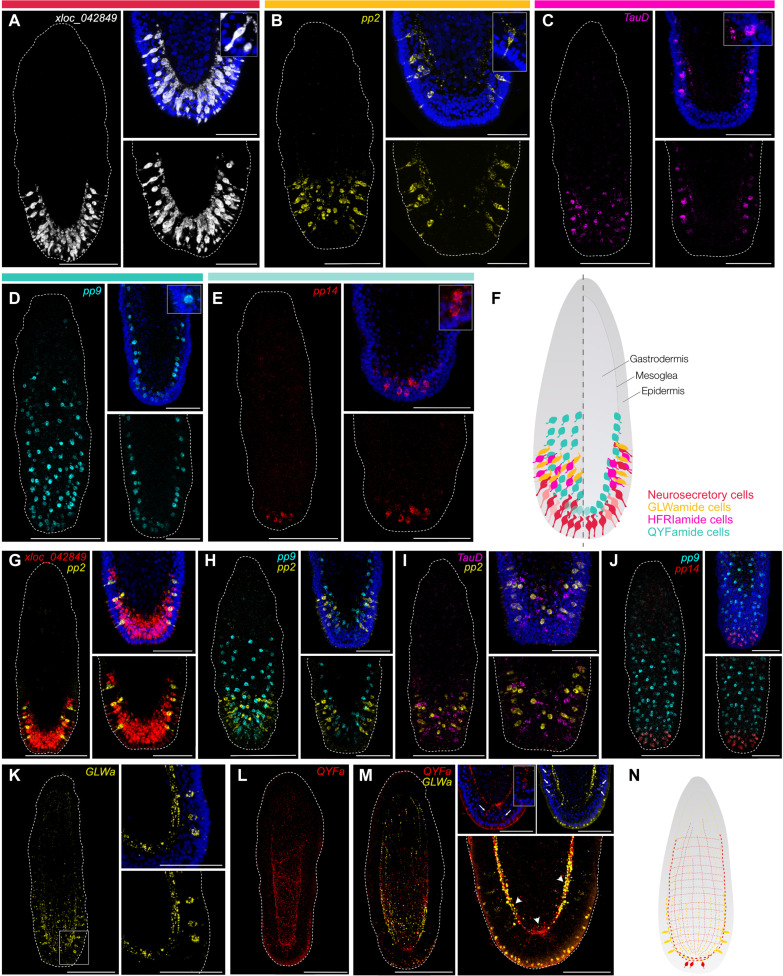
Distinct morphologies of specialized aboral cell types in the *Clytia* planula. (**A** and **B**) FISH for xloc_042849 (white) and pp2 (yellow). (**C** to **E**) HCRs for TauD (magenta), pp9 (cyan), and pp14 (red). In all panels (A) to (E), magnifications of the aboral ends are shown in the right panels; blue in upper right panels corresponds to Hoechst staining. Insets show examples of individual cell bodies. (**F**) Schematic of aboral cell type distribution in the *Clytia* planula: surface view (left) and medial view (right). The color code of the cells matches the bars above panels (A) to (E). (**G** to **J**) Double HCRs for xloc_042849 (red), pp2 (yellow), pp9 (cyan), TauD (magenta), and pp14 (red). Magnifications of the aboral ends are shown in the right panels. Blue staining in upper right panels of (G) to (J) corresponds to Hoechst staining. (**K** to **M**) Neural networks stained with anti-GLWa (yellow) and anti-QYFa (red). Right panels in (K) show a magnification of the zone outlined in the left panel. Right panels in (M) show a magnified view of the aboral pole, with the upper panels corresponding to individual channels combined with Hoechst staining (blue). The inset shows an individual cell body. Arrowheads in (M) indicate the aboral nerve plexus, while arrows point to individual cell bodies. (**N**) Schematics of the GLWa and QYFa neural networks and cell bodies in the *Clytia* planula. All images are maximum intensity projections of confocal *Z*-stacks. Left panels in (B) to (D), (H), and (K), and panels (E), (I), and (J) include both surface and medial stacks; Right panels in (B) to (D), (H), and (K), and panels (A),(G), (L), and (M) include only medial stacks. Planulae all oriented with the oral pole uppermost. Scale bars, 100 μm in full planulae; scale bars, 50 μm in aboral magnifications.

Neurosecretory cells (Cluster 14) were characterized by high expression of many PF1 family secreted proteins, as well as specific expression of two *Clytia*-specific astacin family extracellular metalloproteases (Fig. 2D). ISH revealed dense populations of these cells in the epidermis of the aboral tip of the planula, with a flask-shape morphology and tapered apical section, typical of secretory cells (Fig. 2E). Fluorescence ISH (FISH) for a marker gene combined with Hoechst staining (Fig. 3A) further revealed basally positioned nuclei, in contrast to the apical nuclei of the neighboring epithelial-muscular cells. Basal projections were detected connecting to the apical nerve plexus (Fig. 3A), suggesting potential signal exchange with other neural cells.

The GLWamide cells (cluster 23) express two precursor genes for GLWamide family neuropeptides, pp2 and pp11 (*51*). GLWamides have strong settlement and/or metamorphosis inducing activity in planulae from several cnidarian species including *Clytia* (*14*, *52*). This cluster also contains cells expressing a gene encoding multiple repeats of the amino acid motif “IPYFAN” followed by dibasic cleavage-like sites, although without the glycine typical of amidated neuropeptides. The GLWamide cells also express five GPCRs, none of which are detected in other clusters, including a class B GPCR of “adhesion type” and the aborally enriched metabotropic receptor GABA_B_R-like (Fig. 2D). ISH revealed a ring of sensory-like cells coexpressing pp2 and GABA_B_R-like in the epidermis around the aboral pole (Fig. 2E and fig. S8). These cells have club-shaped bodies with apical processes extending toward the outer surface and basal neurites connecting to the apical nerve plexus (Fig. 3B). Immunostaining with an anti-LWamide antibody revealed a concentration of neurite processes at the apical end of the larva, with projections extending longitudinally toward the oral end (Fig. 3, K and M).

HFRIamide cells (cluster 25) are characterized by expression of high levels of a gene that we identify as a putative precursor for a previously undescribed neuropeptide, HFRIamide. The predicted amino acid sequence contains a signal peptide and HFRI sequence followed by a glycine, suggesting amidation (*53*), preceding the dibasic cleavage site. The characteristic protein sequence, including a signal peptide and cleavage site, is conserved in other cnidarian species from both Medusozoa and Anthozoa (fig. S9). Other markers for this cluster include the two aborally enriched TauD genes, as well as the aborally enriched vGAT-like and BGT-1–like transporters (Fig. 2D). These cells show a similar sensory-like morphology and distribution to the GLWamide cells, forming a ring at the surface of the aboral epidermis (Fig. 3C). Double in situ hybridization chain reaction (HCR), however, shows distinct cell populations (Fig. 3I).

QYFamide cells (cluster 20) are defined by the high and specific expression of the neuropeptide precursor gene pp9 (*54*) predicted to generate pyroGluYFamide as well as other amidated neuropeptides. Their transcriptome also shows enriched expression of a nitric oxide (NO) synthase. In addition, specific markers include two contactin-associated proteins, a zinc finger transcription factor ortholog to the *Nematostella* Prdm14d, shown to be involved in nonectodermal neurogenesis (*55*), and several receptors, including a ligand-gated ion channel with homology to a GABA_A_R subunit, and two class A GPCRs, one of which is very divergent (Fig. 2D). QYFamide cells are located in a zone between the aboral tip and the medial region of the planula. Another neuropeptide precursor, pp14 (likely generating the peptide GPGSRFLFamide) (*54*), shows expression in a subpopulation of the QYFamide cells, concentrated at the aboral tip (Fig. 2E). pp9-expressing cells have a ganglionic-like appearance, distinct from the other neural aboral cell types and characterized by large cell bodies positioned at the base of the epidermis, just above the mesoglea—the basal lamina separating the epidermis from the gastrodermis (Fig. 3, D and E). Double HCR for pp9 and pp14 confirmed their coexpression in an aboral subpopulation of cells in the neural plexus region (Fig. 3J). Staining with an anti-pyroGluYFamide antibody showed dense concentration in the aboral nerve plexus and nerve fibers extending the length of the planula body in the mesoglea layer (Fig. 3L). A few individual cell bodies associated with the apical nerve plexus were also stained (Fig. 3M). Their neurites are organized as an array of perpendicular bundles, in contrast to the GLWamide fibers that run only longitudinally along the planula cell body (Fig. 3M). The observed organization of these ganglionic-type QYFamide cells suggests that they regulate contraction of the myofibrils that extend from the basal sides of the ectodermal and gastrodermal epithelial cells in longitudinal and circular directions, respectively, to control the planula shape.

### Aboral cell types of two coral planula larvae

**Fig. 4.**
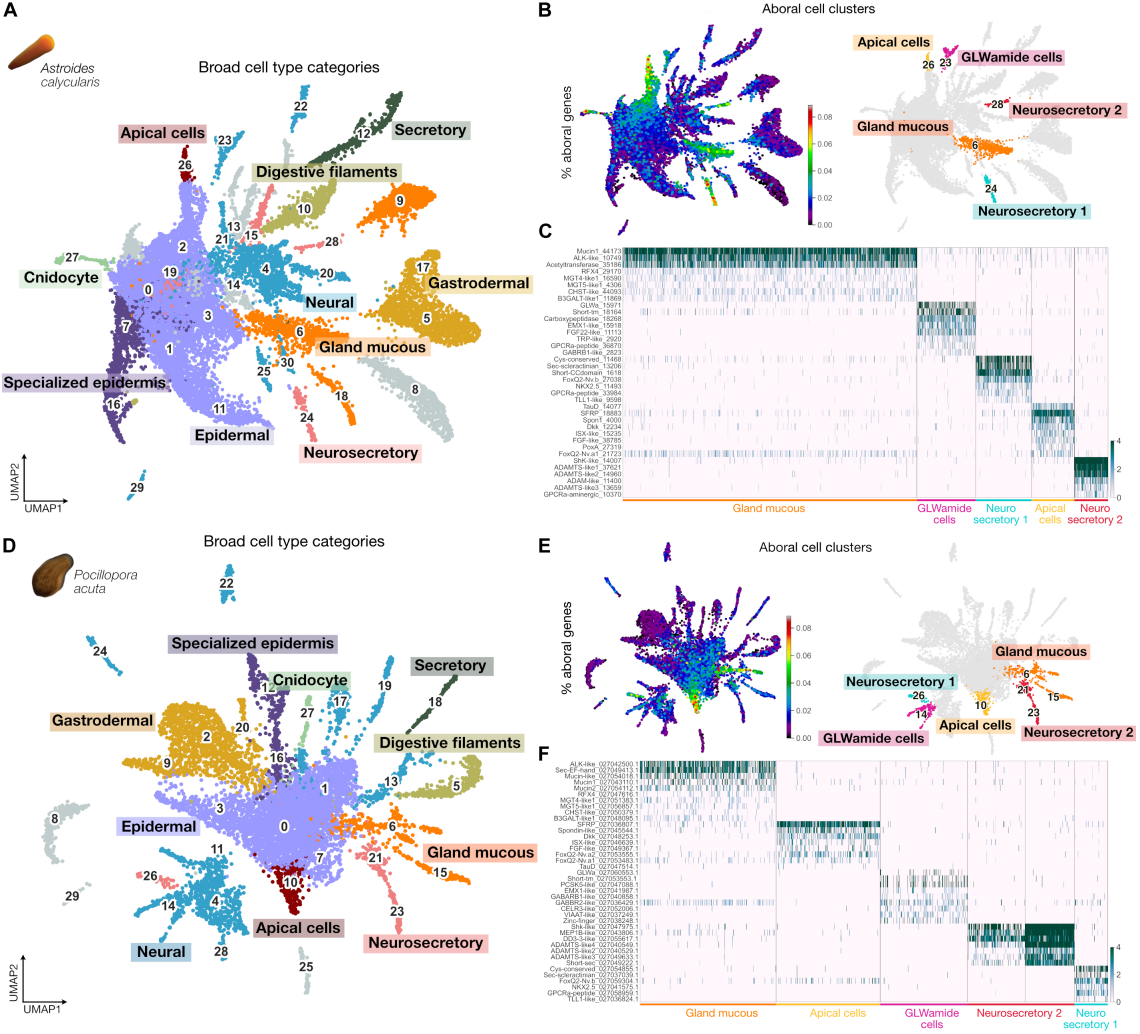
Transcriptomic characterization of aboral cell types in *Astroides* and *Pocillopora* planulae. (**A** and **D**) UMAP representation of *Astroides* and *Pocillopora* planula cells respectively labeled by cell type classes: epidermal, specialized epidermis, gastrodermal, digestive filaments, neural, secretory, gland mucous, neurosecretory, apical cells, and cnidocytes. Gray clusters have not been annotated. (**B** and **E**) Aborally enriched cell clusters in the *Astroides* and *Pocillopora* planula cell atlases, respectively. On the left, UMAP of *Astroides*/*Pocillopora* planula cells, with cells colored on the basis of the percentage of expression of aborally enriched genes. Warmer colors indicate high proportions of aboral gene counts relative to the total gene counts in each cell. On the right, *Astroides*/*Pocillopora* planula cell atlas with aboral cell clusters highlighted in distinct colors. *P* values for aboral enrichment significance (Fisher’s exact test) were as follows: gland mucous, 1.75 × 10^−06^; GLWamide cells, 0.71; neurosecretory 1, 3.38 × 10^−22^; neurosecretory 2, 8.97 × 10^−05^; apical cells, 1.38 × 10^−30^ for *Astroides*; gland mucous_6, 2.71 × 10^−05^; gland mucous_15, 0.09; GLWamide cells, 0.73; neurosecretory 1, 5.06 × 10^−05^; neurosecretory 2_21, 6.57 × 10^−04^; neurosecretory 2_23, 0.71; apical cells, 8.92 × 10^−20^ for *Pocillopora*. (**C** and **F**) Heatmap of marker genes for each *Astroides*/*Pocillopora* aboral cell cluster, with cell type annotation labels and colors corresponding to (B) and (E), respectively (complete gene list in table S3). Gene names are based on orthology assignments or Pfam domains when orthology is unclear. Trailing numbers are unique gene identifiers from this project.

For *Astroides* and *Pocillopora*, we dissociated planula collected from natural releases and carried out three rounds of 10x Genomics scRNA-seq cell capture for each species. After analysis, we retained 18,964 *Astroides* and 9733 *Pocillopora* planula cells expressing a total of 21,952 and 17,112 genes, respectively. Using Leiden clustering, we resolved 31 clusters in the *Astroides* planula cell atlas and 30 clusters in the *Pocillopora* planula cell atlas with distinct expression signatures (fig. S10). Clusters were grouped into 10 broad cell classes, consistent with previously published cell atlases of anthozoan larvae [*Stylophora pistillata* (*56*) and *N. vectensis* (*57*, *58*)]: epidermal, specialized epidermis, gastrodermal, digestive filaments, neural, secretory, gland mucous, neurosecretory, apical cells, and cnidocytes (Fig. 4, A and D, and figs. S11 and S12).

By integrating our *Astroides* and *Pocillopora* aboral transcriptomes with the planula scRNA-seq data, we identified five aboral cell clusters for each species showing equivalent transcriptomic signatures: three cell clusters with a secretory signature (gland-mucous, neurosecretory 1, and neurosecretory 2), one neural cluster (GLWamide cells), and one epidermal-like cluster (apical cells) (Fig. 4, B and E).

### Transcriptomic and morphological characterization of the coral planula aboral cell types

*Astroides* and *Pocillopora* planula scRNA-seq clustering shows strong similarity to the *Astroides* dataset containing a larger number of cells. We used *Astroides* planulae, which lack symbionts, for morphological characterization of the five aboral cell types using HCR of selected marker genes and antibody staining.

**Fig. 5.**
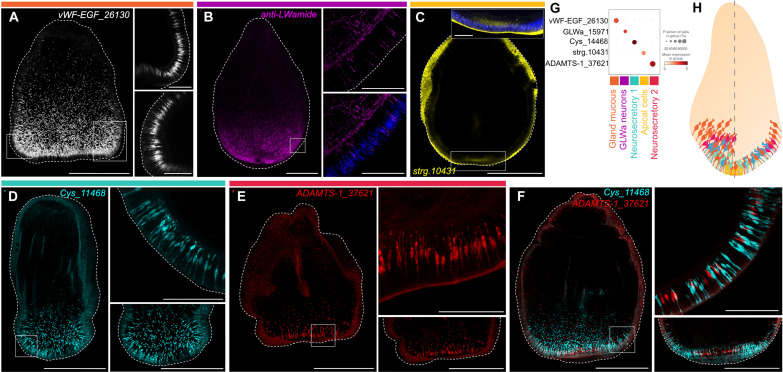
Morphology of aboral cell types in the *Astroides* planula. (**A** and **C** to **E**) HCRs for vWF-EGF_26130 (white), strg.10431 (yellow), Cys_11468 (cyan), and ADAMTS-1_37621 (red). (**B**) Anti-LWamide staining (magenta). Blue staining in the lower right panel in (B) and upper right inset in (C) shows Hoechst staining. (**F**) Double HCR for Cys_11468 (cyan) and ADAMTS-1_37621 (red). Squares in (A) to (F) outline the areas shown in higher magnification to the right. All images are maximum intensity projections of confocal *Z*-stacks. Scale bars, 500 μm in full planulae and 100 μm in the high-magnification panels. (**G**) Dot plot of expression of the marker genes shown in (A) to (F) across the aboral cell clusters. (**H**) Schematics showing the distribution of aboral cell types in the *Astroides* planula. The left part corresponds to a surface view, and the right part corresponds to a medial section. The color code of the cells matches the bars above the (A) to (E) panels and the annotation labels in (G).

The gland-mucous cell transcriptome is characterized by the expression of mucins and different types of enzymes with potential roles catalyzing posttranslational modifications to glycoproteins and glycans including two α-1,3-mannosyl-glycoprotein 4-β-*N*-acetylglucosaminyltransferase–like proteins, two β-1,3-galactosyltransferase–like proteins, one α-1,6-mannosylglycoprotein 6-β-*N*-acetylglucosaminyltransferase–like protein, and one carbohydrate sulfotransferase–like protein. These cells also specifically express an ALK-like tyrosine kinase receptor and the RFX4 transcription factor, both of which are also markers for the mucous cells in *Clytia* (*46*) and the gland-mucous cells in the *Nematostella* planula (*58*) (Fig. 4, C and F). HCR for a gland-mucous cell marker gene shows staining in club-shaped cells densely covering the whole aboral epidermis (Fig. 5A). They bear apical extensions that reach the surface, consistent with a secretory function.

The two “neurosecretory” clusters show expression of neural markers, including ELAV and the transcription factor AshA (*58*, *59*), and several genes potentially encoding secreted proteins. The “neurosecretory 1” cells are characterized by high expression of two short secreted proteins. One is specific to Anthozoa and, like the PF1 secreted proteins expressed in *Clytia* neurosecretory cells, contains conserved cysteines (fig. S2). The other is exclusively found in other scleractinian coral species (see table S3). In both coral species, these cells also specifically express a class A GPCR of peptide-binding type and a metalloprotease related to the Tolloid family, involved in cleaving proteins in the extracellular matrix. They also express two transcription factors: Nkx2.5, identified as apical enriched in *Nematostella* and two *Acropora* species (*27*), and a forkhead transcription factor that is a paralog of the aboral FoxQ2a in *Nematostella* (Fig. 4, C and F).

The “neurosecretory 2” cells are characterized by the high expression of a cysteine-rich secreted protein with an ShK-like domain, characteristic of the potassium channel–blocking toxin found in the sea anemone *Stichodactyla helianthus* (*60*). These types of domains have also been identified in cnidocyte venom components and in toxin-like neuropeptides in *Nematostella* (*61*–*63*). This neurosecretory type also shows specific expression of four metalloproteinases of the ADAM family, three of which also detected as significantly enriched in the aboral end (Figs. 1G and 4, C and F). The two neurosecretory cell types show a very similar secretory-like morphology to the gland mucous cells as shown by HCR (Fig. 5, D and E). They are also concentrated at the apical tip, although in lower numbers. Double HCR for marker genes of each neurosecretory type confirmed expression in distinct populations (Fig. 5F).

The aboral neural cell cluster is defined by the expression of the GLWamide neuropeptide precursor gene. In both *Astroides* and *Pocillopora*, the transcriptome of these GLWamide cells shows specific expression of neural-related genes, including enzymes involved in the processing of propeptides into their biologically active form: a carboxypeptidase in *Astroides* and a proprotein convertase in *Pocillopora* (Fig. 4, C and F). Among the few genes that show cluster-specific expression is a neurotransmitter-gated ion channel with similarity to GABA_A_Rs (fig. S13) and a homeobox transcription factor EMX-like. Staining with an anti-LWamide antibody revealed a dense nerve plexus at the aboral end of the planula, associated with individual cell bodies within the epidermal layer in an aboral-lateral zone (Fig. 5B). No cell bodies were detected at the apical tip where neurosecretory cells are more abundant.

The coral planula scRNA-seq analysis revealed one aboral cell type clearly distinct from any of those in the *Clytia* planula, the cluster that we term “apical cells.” This cluster expresses the highest proportion of aborally enriched genes (Fig. 4, B and E). Its transcriptomic signature is closely related to the epidermal clusters as shown by the expression of shared marker genes, including a tropomyosin-like and the transcription factor Sox3, expressed in the ectoderm of *Nematostella* (*58*). Among specific apical cell cluster markers are many of the genes forming the gene regulatory network directing the formation of the apical organ in the *Nematostella* planula. These include known aborally/anteriorly expressed genes required for the development of the apical organ, including Six3/6, FoxQ2a (*17*), FGFa1 (*28*), and ISX-like (*6*); and other apical markers shown to have specific expression in the apical domain of *Nematostella* planula including Frizzled 5/8, Dickkopf-like, SFRP, Nkx3, Tbx4-5, and BMP1-like5 (*27*, *30*). For some of these genes, distinct expression patterns have been observed in one of the two cell types forming the apical organ of *Nematostella*—the apical tuft cells and the larva-specific neurons (*6*, *7*, *57*). However, all apical organ markers are found coexpressed in the same apical cell cluster of the coral planulae (fig. S14). Comparison of planula data from another scleractinian coral, *S. pistillata*, identified a cluster directly corresponding to the *Astroides* and *Pocillopora* apical cells (*27*, *56*). Together, these observations indicate the homology of the coral apical cells to the *Nematostella* apical organ. A cell type with the “apical cell” signature possibly emerged before the divergence of Actiniaria and Scleractinia, and the subdivision of the apical organ into two distinct cell types evolved subsequently in actinarian sea anemones.

Another notable marker for the coral planula apical cells is a gene containing a TauD domain (Fig. 4, C and F), orthologous to the TauD gene identified in the apical domain of *Nematostella* (*30*) and closely related to the two *Clytia* TauD genes expressed in the HFRIamide cells (fig. S4). HCR for a coral apical cell marker gene in *Astroides* larvae showed signal in a localized region of the epidermis at the most aboral tip (Fig. 5C). The cell bodies are positioned slightly below the aboral surface, their nuclei being less densely packed and positioned deeper than the surrounding epithelial cells (Fig. 5C). This pattern indicates that specialized epidermal cells at the aboral tip in these coral larvae have a distinct structure.

### Taurine inhibits settlement in both medusozoan and anthozoan planulae

The cnidarian TauD-containing genes emerged from our analyses as a notable common feature of the planula aboral ends, albeit expressed in distinct cell types. The two *Clytia* genes are expressed in the HFRIamide cells, while in the coral larvae, a closely related gene is expressed in apical epidermal-like cells lacking a neural signature. The HFRIamide cells in *Clytia* also express the vGAT-like and BGT-1–like transporters, potentially able to mediate uptake of GABA/glycine/taurine–related amino acids. The expression of TauD orthologs in specialized cells at the aboral end of other anthozoan planulae (*27*, *30*) suggests a shared and possibly ancestral role across cnidarian larvae. Although the molecular function of these particular TauD proteins remains to be determined, one intriguing possibility is that they are involved in regulating settlement via catabolism of taurine. Complex prediction using the TauD protein from *Clytia* with taurine, 2-oxoglutarate (α-ketoglutarate), and Fe^2+^ as inputs to boltz-1 (*64*) placed the ligands in essentially the same positions as that found in the *Escherichia coli* crystal structure of TauD (*65*), with no obvious steric clashes (fig. S15).

Taurine is an amino sulfonic acid widely implicated in many processes of cell metabolism, including osmoregulation, calcium modulation, and antioxidation (*66*, *67*). In the mammalian brain, it can act as an agonist at receptors of the GABAergic and glycinergic neurotransmitter systems (*45*), so it is a potential ligand for the GABA_B_R-type receptors expressed in the GLWamide cells. It has been reported to function as a fast neurotransmitter in the scyphozoan “lion’s mane” jellyfish *Cyanea capillata* (*68*). Furthermore, a study in hydrozoan larvae has implicated taurine in maintaining the planula state (*69*).

**Fig. 6.**
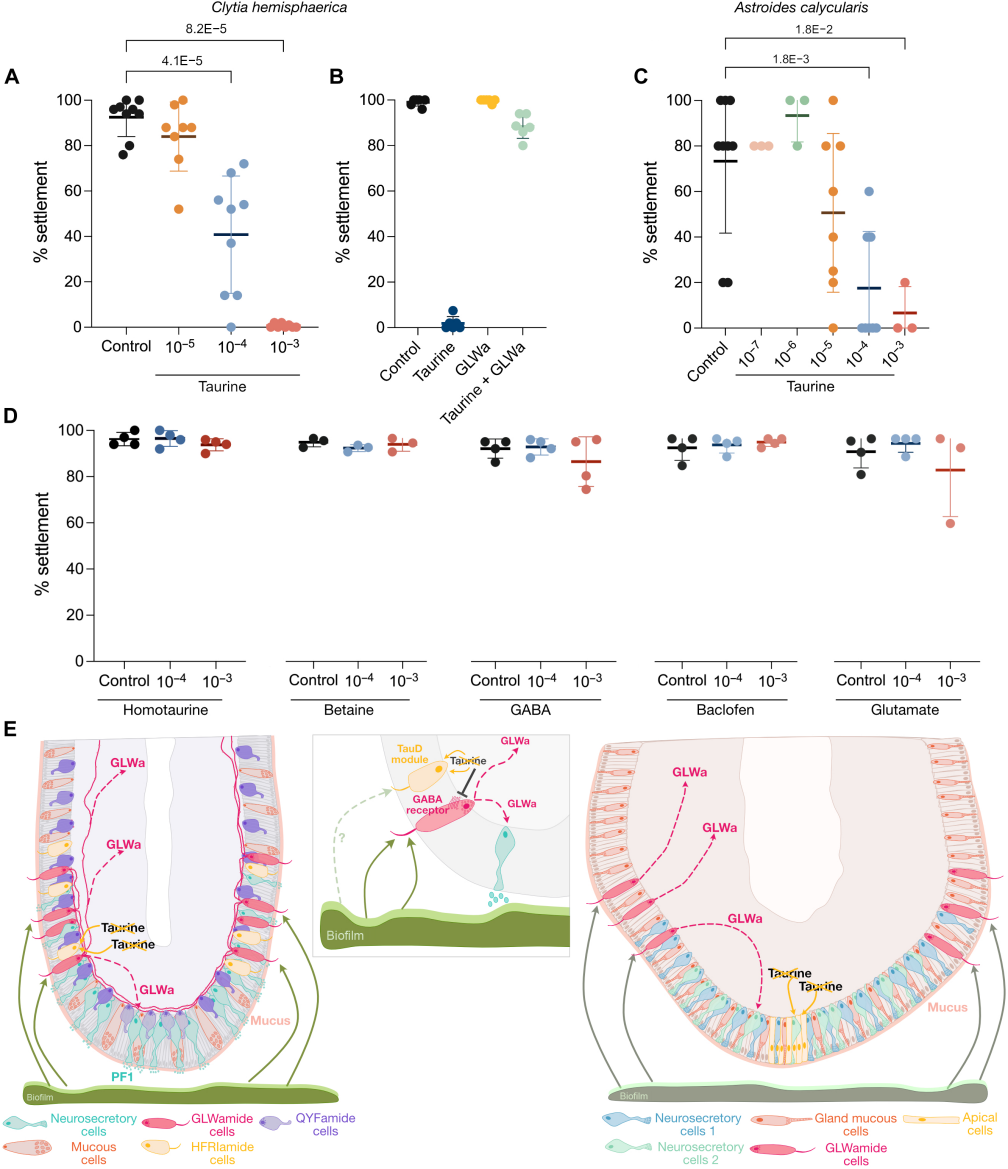
Taurine inhibition of settlement in hydrozoan and coral planulae. (**A** and **B**) Effect of taurine treatment on *Clytia* settlement. Plots show means ± SD, with concentration units expressed in molarity (M). Each dot represents the percentage of larvae settled in an individual well after overnight treatment (100 or 50 planulae per condition). *P* values are indicated for significant differences with respect to the control condition (Mann-Whitney *U* test). (B) Combined treatment with taurine (10^−3^ M) and GLWamide (10^−6^ M). Eight independent experiments were done for taurine and six for taurine + GLWamide (see raw treatment data in table S4). (**C**) Effect of taurine treatment on *Astroides* settlement. Plots show means ± SD, with concentration units expressed in molarity (M). Each dot corresponds to the percentage of larvae that had settled in an individual well on the first day >50% of control larvae settled (five planulae per condition). Eight independent experiments were carried out for taurine at 10^−4^ and 10^−5^ M and three for taurine at 10^−3^, 10^−6^, and 10^−7^ M (see raw treatment data in table S4; see fig. S16 for settlement plots across all days of treatment). *P* values are indicated for significant differences with respect to the control condition (Mann-Whitney *U* test). (**D**) Effect of other molecules on *Clytia* settlement. Plots show means ± SD. Each dot represents the percentage of larvae settled in an individual well after overnight treatment (100 or 50 planulae per condition). Four experiments for homotaurine, GABA, baclofen, and glutamate and three for betaine (see raw treatment data in table S4). (**E**) Proposed model of the cells and molecules involved in cnidarian larval settlement. The schematic shows the aboral end of *Clytia* (left) and coral planula (right), with an inset illustrating the proposed regulatory interactions between HFRIamide cells and GLWamide cells within the *Clytia* planula.

We tested the effect of taurine on larval settlement using *Clytia* in the presence of natural biofilms and *Astroides* in the absence of active aeration, conditions that both promote settlement. More extensive testing was possible with *Clytia*, for which larvae are readily available from laboratory cultures. Exogenous taurine impaired settlement in both species—with nearly 100% inhibition observed when planulae were incubated in 10^−3^ or 10^−4^ M taurine (Fig. 6, A to C). Using *Clytia*, we could show that the taurine inhibition could be overridden by treatment with GLWamide, placing inhibition by taurine upstream of GLWa secretion (Fig. 6B).

In the hydroid *Hydractinia echinata*, taurine and molecules with *N*-methyl groups, such as *N*-methylpicolinic acid (homarine), *N*-methylnicotinic acid (trigonelline), and *N*-trimethylglycine (betaine), were detected in high concentrations in the larvae, the levels decreasing upon settlement induction (*70*), and inhibit metamorphosis when present in micromolar concentrations (*71*, *72*). Other structurally similar molecules, including GABA and the GABA agonist baclofen, inhibit the planula-to-polyp transition in the sea anemone *Nematostella* (*41*). In our tests using *Clytia* planulae, neither homotaurine, betaine, GABA, baclofen, nor glutamate impaired settlement when present at the same concentrations used for taurine treatments (Fig. 6D), indicating that, at least in *Clytia*, taurine is a specific settlement inhibitor. One possible interpretation of these findings is that specialized aboral cells in cnidarian planulae contribute to regulating settlement by decreasing taurine levels locally, a hypothesis discussed in more detail below.

## DISCUSSION

We have identified shared molecular and cellular features of planula aboral domains of the hydrozoan *C. hemisphaerica* and the scleractinian corals *A. calycularis* and *P. acuta*. As illustrated in Fig. 6E and fig. S17, two broad cell type classes—the secretory-like and the neural-like—have a similar arrangement at the aboral ends of both types of planulae.

The secretory cell types all show flask-shaped morphology with a tapered apical section extending toward the surface to secrete molecules into the environment, as previously deduced from histology (*2*, *73*, *74*). Neurosecretory cell types in both *Clytia* and coral planulae are characterized by high expression levels of taxon-restricted, short secreted proteins that share similar features, including conserved cysteines indicative of disulfide bonding. These include the PF1 secreted proteins in *Clytia*, and a distinct secreted protein with a unique 3D structure in one of the neurosecretory cell types of the coral planulae. The function of these highly abundant proteins is unknown. They could potentially have antimicrobial peptide activities, but another possibility is that, together with the secreted mucins, they help mediate larval adhesion to the substrate. A family of small, cysteine-rich secreted proteins is involved in forming a specialized attachment holdfast in mussels, facilitating cross-linking within the cuticle (*75*). Small proteins with cysteines in conserved positions have also been identified in barnacle cement (*76*), and multiprotein secreted adhesion systems are similarly responsible for the adhesion of *Drosophila* pupae (*77*). In each of these examples, the genes involved are species specific.

Cnidarian larvae also bear another specialized type of secretory cell, the mucous cells, concentrated in aboral and lateral regions. Mucous secretion has been implicated in many facets of cnidarian biology including innate immunity and management of associated microbial communities (*78*). Other roles for mucus secreted by the larva include protection of the larvae against suspended particles (*79*) and potentially also promotion of settlement by slowing down larval swimming at substrate interfaces, promoting adhesion, and/or enhancing bacterial biofilm production (*73*, *80*).

GLWamide cells in both *Clytia* and *Astroides* planulae surround the aboral pole and show the same sensory-like morphology and associated neurite network. Enrichment of GLWamide immunoreactive cell bodies and their neurites has been observed in aboral domains of various medusozoan planulae (*3*, *8*, *9*, *81*), and GLWamide-expressing cells have been detected across an aboral-central domain of the epidermis in the coral *Acropora* planula (*5*). GLWamide has been widely implicated in triggering planula cells to undergo metamorphosis, being released downstream of unknown sensory inputs (*69*). The shared expression of GPCRs and ligand-gated ion channels showing homology to GABA receptors in aboral cells of *Clytia* and coral planulae is thus of note. We identified a GABA_B_R-like expressed in *Clytia* and *Pocillopora* GLWamide cells and a GABA_A_R-like in both corals *Astroides* and *Pocillopora.* Both receptors come from gene families expanded in cnidarians, so it is possible that ligand binding specificity has altered. Treatment with GABA_B_R agonists has been shown to inhibit metamorphosis in *Nematostella* (*41*). Together, these findings suggest a conserved role of GABA-like receptors in the planula nervous system, potentially involved in the regulation of the settlement response, although not necessarily via GABA. In our assays, neither GABA nor its agonist baclofen had any effect on settlement in *Clytia*. This suggests that a distinct ligand may bind the “GABA-like” receptors in *Clytia.*

A number of observations point to taurine as the substrate of the TauD proteins expressed aborally in *Clytia* and coral planulae. First, our findings and others have directly implicated taurine in cnidarian planula biology (*4*, *27*, *30*, *70*). Furthermore, cnidarian larvae for which appropriate data are available show aboral expression of genes encoding Pfam TauD domains (*27*, *30*). These take their name from *E. coli* taurine dioxygenase, although there is no direct evidence that taurine is the endogenous substrate in cnidarians. Two human genes, BBOX1 (γ-butyrobetaine dioxygenase) and TMLHE (trimethyllysine dioxygenase), encode a TauD domain. These are both involved in carnitine biosynthesis, but both have more plausible cnidarian orthologs than the genes we have identified as aborally enriched. In *Clytia* planulae, the aboral TauD-expressing cells are neural and coexpress genes potentially involved in taurine uptake, including vGAT-like and BGT-1–like transporters. The same TauD-encoding genes are also specifically expressed in neurons in the *Clytia* medusa stage (*54*), and it is of note that taurine is an agonist of GABA receptors, close homologs of which we find in GLWamide cells. Last, we have shown that taurine has a common effect in inhibiting settlement in planulae of both *Astroides* coral and *Clytia*. Taking these observations together, we find it reasonable to hypothesize that taurine is a candidate substrate of the TauD proteins.

Putting together the different lines of evidence concerning the aboral TauD protein expression and GLWamide cells, we propose one possible model for settlement regulation (Fig. 6E). High levels of taurine present in larval tissue premetamorphosis (*70*) inhibit spontaneous settlement by binding to metabotropic GABA_B_R-type GPCRs and/or ionotropic GABA_A_R-type receptors in the plasma membranes of GLWamide cells, thereby preventing GLWamide secretion. To allow settlement, aboral cells specialized in the uptake and catabolism of taurine—the HFRIamide cells in *Clytia* and the apical cells in the corals—would act to deplete extracellular taurine locally, thus relieving the inhibition on GLWamide cells and permitting secretion of GLWamide neuropeptides. This mechanism may have been selected during the evolution of planulae to regulate settlement, potentially preventing this process either when the planula is not physiologically ready and/or because environmental conditions are unsuitable. The taurine catabolism genes are expressed in distinct specialized aboral cell types in hydrozoan and coral planulae as well as in the planula of the sea anemone *Nematostella* where it is expressed in the apical organ cells (*30*). The current situation may have evolved from an ancestral broader expression of taurine catabolism–related genes in an aboral domain, for example, under regulation of aboral domain transcription factors such as Six3/6 or FoxQ2a (*17*). Throughout the evolution of planulae across diverse species, the TauD proteins may have been selectively retained in different cell types within the aboral end as they diversified independently across species. Alternatively, the involvement of these TauD proteins in settlement regulation may have converged independently in different cnidarian groups, accounting for their expression in distinct cell types in *Clytia* and corals.

The mechanism proposed in Fig. 6E, in which local lifting of taurine-mediated inhibitory action around GLWamide-secreting cells allows cnidarian larvae to respond to environmental cues, integrates our findings but is obviously speculative. In any case, settlement induction is likely to require multimodal input (*69*, *82*) acting on sensory cells such as GLWamide cells, HFRIamide cells, and potentially, aboral Nk2d cells in *Clytia*. NO produced by the *Clytia* QYFamide cells is a candidate settlement-inducing signal, these ganglionic-type cells of the aboral neural plexus potentially integrating signals from distinct secreted molecules emitted by the sensory cells. NO is implicated, positively or negatively, in the regulation of settlement and metamorphosis in diverse marine invertebrates (*83*–*89*). Overall, this study provides the foundations for a full cellular and molecular understanding of the settlement response in cnidarians through functional analysis of the various candidate receptors and signaling molecules identified in these distinct aboral cell types.

## MATERIALS AND METHODS

No animal experimentation performed required ethics approval.

### Larva collection

*Clytia* larvae were obtained from fertilizations of laboratory Z strain adults (*40*). Gametes collected following light-induced spawning of male and female medusae were mixed, and cleaving embryos were transferred into small glass dishes filled with Millipore-filtered artificial seawater (MFSW; Red Sea Salt brand) (*14*). Embryos were left to develop at temperatures of 17° to 18°C. Development to the larval stage is rapid and reproducible in the laboratory; once the two body cell layers are fully established and all cell types present, at around 48 hpf, the larva is already able to settle in response to biofilm cues.

*Astroides* larvae were obtained following release from wild colonies. Fragments of adult female colonies were collected from three sampling sites on the island of Ischia, with permission for larva sampling of *A. calycularis* granted by the Marine Protected Area “Regno di Nettuno” [Sant’Angelo (40°41′31.1″N, 13°53′35.0″E), San Pancrazio (40°42′6.30″N, 13°57′17.8″E), and Punta Vico (40°45′33.4″N, 13°53′1.51″E)], a few days before the full moon of June 2021, 2022, and 2023. They were maintained in a 30-liter tank with water motion provided by a NEWA mini 606 pump and kept under dim-light conditions. Larvae were observed by transparency in the gastrovascular cavity and tentacles of the female colonies (*90*). Mature larvae fully developed inside the mother colonies were released 1 to 2 days after collection. Larvae were transferred into 300-ml plastic bottles filled with seawater and transported to the Villefranche laboratory in less than 24 hours. At the laboratory, larvae were kept in a temperature-controlled room at 24°C in 10-liter plastic containers filled with seawater and constant aeration. We consider these batches of larvae to be competent to settle, because in the absence of aeration, spontaneous settlement and metamorphosis were observed in the tanks. Larvae collected in June 2021 were used for generating the aboral/oral transcriptomic data and the scRNA-seq data. Larvae collected in June 2021, 2022, and 2023 were used for immunostaining, ISHs, and settlement assays.

*Pocillopora* larvae were collected following release from *P. acuta* (formerly assigned to lineage type β in the *Pocillopora damicornis* species complex) parent colonies, long-term cultured and asexually propagated by fragmentation at the Océanopolis Brest aquarium facility (six tagged colonies, ~15 to 20 cm diameter, corresponding to six phenotypes of the same genotype). Brooding colonies were placed individually in plankton net (200-μm mesh)–fitted polyvinyl chloride cylinders each evening and transferred back to the holding tank the following morning for a few successive days before the full moon. Night-released planula larvae were buoyant and actively swimming, hosting endosymbiotic *Cladocopium* sp. dinoflagellates (Symbiodiniaceae family) vertically transmitted from each parent colony (*91*). They were collected with a glass Pasteur pipette, transferred to 300-ml polyethylene bottles filled with 0.2 μm–filtered seawater (FSW) (separate batches per each parent colony), and transported to the laboratory within 2 days from release (*15*, *91*). FSW was renewed daily (50% of the total volume) and aerated using orbital agitation (Infors incubator, 25 rpm, MCAM laboratory, Paris) at 24°C under a 12-hour day:12-hour night photoperiod (~70 μmol photons/m^2^ per second, white and blue light) or by gentle air bubbling (LBDV laboratory). Larvae were sorted daily by developmental stage under a stereomicroscope to remove early metamorphic (disk-flattened) stages, retaining only the elongated, swimming planulae. Larvae released from individual colonies “C3” and “C5” around the time of the full moon of February 2020 were used for the generation of the aboral/oral bulk RNA-seq data. Larvae released from the “C3” colony around the full moon of December 2022 (6 and 7 December 2022) and from colony “C6” around the full moon of February 2023 (5 and 6 February 2023) were used for the generation of scRNA-seq data.

### Dissection of aboral/oral ends

Aboral and oral ends of *Clytia* larvae (52 hpf) were separated manually in MFSW using handmade tungsten wire loops and 2% agarose/MFSW–lined plastic petri dishes. Larvae were anesthetized by placing the dish on ice for 10 min and bisected under a stereomicroscope. For *Astroides* and *Pocillopora*, aboral and oral ends were separated using microdissection scissors under a stereomicroscope. Dissected tissues were transferred to lysis buffer (Ambion, RNAqueous MicroKit), vortexed, immediately snap frozen in liquid nitrogen, and stored at −80°C until mRNA extraction. *Clytia* larvae were dissected in rounds of maximum 30 min, pooled and placed in lysis buffer, and snap frozen. *Astroides* and *Pocillopora* larvae were dissected for 5 to 10 min, immediately placed in the lysis buffer, and snap frozen.

### Bulk RNA-seq and differential gene expression analysis

For RNA extraction, tissue samples from several aboral/oral dissections were pooled to ensure sufficient RNA yield for sequencing. For the *Clytia* samples, RNA was extracted from 321 aboral halves, 314 oral halves, and 140 uncut larvae for biological replicate 1 and 320 aboral halves, 317 oral halves, and 140 uncut larvae for biological replicate 2. For the *Astroides* samples, RNA was extracted from 20 oral and aboral halves and 10 uncut larvae for each of the two biological replicates. For the *Pocillopora* samples, RNA was extracted from four aboral halves and two oral halves for biological replicate 1 and eight aboral halves and six oral halves for biological replicate 2. Because *Clytia* larvae are much smaller than those of the corals, more larvae were pooled to achieve the equivalent quality and quantity of purified mRNA, allowing equivalent sequencing depth. Note that our strategy was to use these oral versus aboral bulk mRNA comparisons primarily as an entry point to identify and explore thoroughly aboral cells through scRNA-seq. Total RNA was isolated from each sample using the RNAqueous Total RNA Isolation Kit (Ambion, Thermo Fisher Scientific). Treatment with deoxyribonuclease I (Q1 DNAse, Promega) for 20 min at 37°C (2 U per sample) was followed by purification using the RNeasy minElute Cleanup kit (Qiagen). The RNA quality of all samples was checked using the Agilent 2100 Bioanalyzer with an Agilent RNA 6000 Nano Kit. Library preparation and sequencing were outsourced (BGI, DNBseq), generating 30 million 100–base pair (bp) paired-end reads per replicate. Gene counts for each sample were obtained by mapping the RNA-seq reads to the corresponding genomes [a chromosome level genome assembly for *Clytia*, an in-house–generated genome for *Astroides* (see below), and the *P. damicornis* genome (*92*) for *Pocillopora*] using STAR (version 2.7.11a) (*93*) with default mapping parameters and the option --quantMode GeneCounts. The gene counts were imported into R, and count matrices integrating the gene counts for each condition were generated. Differential gene expression analyses were performed using the DESeq2 R package (*94*). Batch effects in *Pocillopora* samples, collected from two distinct mother colony phenotypes (see the “Larva collection” section for details), were corrected by incorporating the batch factor into the design formula. PCA and heatmaps were generated using normalized counts obtained with the variance stabilizing transformation (DESeq2 “vst” with blind = F). For the *Pocillopora* dataset, the “removeBatchEffect” function from the limma R package was used to visualize the transformed data with batch variation removed.

### Cell dissociation

For *Clytia*, we prepared single-cell suspensions from planulae obtained from two fertilizations using the same batch of mature jellyfish, one let to develop at 17°C until 52 hpf (late 2-day planulae) and the other until 66 hpf (early 3-day planulae). For each condition, we used ~1000 larvae for a single-cell dissociation, with each dissociation serving as a biological replicate. Larvae were transferred into a 40-μm mesh strainer and placed inside a small plastic petri dish filled with Ca/Mg–free artificial seawater (ASW; 530.5 mM NaCl, 10.7 mM KCl, 3.45 mM NaHCO_3_, and 11.26 mM Na_2_SO_4_, pH 8.0). The larvae were washed several times with Ca/Mg–free ASW to remove any residual cations from the seawater. In the final wash, they were transferred to a small petri dish filled with Ca/Mg–free ASW containing SUPERase·In RNase Inhibitor (40 U/ml; Invitrogen), where they were allowed to incubate for 10 min. Gentle pipetting with a p1000 tip was used to help dissociate the cells.

For the *Astroides* and *Pocillopora* samples, between 10 and 15 larvae were used for each cell dissociation, with each dissociation serving as a biological replicate. Larvae were transferred into a 40-μm mesh strainer and placed in a small plastic petri dish filled with Ca/Mg–free ASW and SUPERase·In RNase Inhibitor (40 U/ml; Invitrogen) for 10 min. Mechanical disruption of the tissue was achieved by gently rubbing the larvae against the mesh using the interior cap of the plunger of a 1-ml syringe. Once the epidermis was disrupted, the tissue was dissociated by gentle pipetting using a p1000 tip.

In all cases, dissociated cells were recovered in 1 ml of low-Ca/Mg-free ASW (460 mM NaCl, 9.93 mM KCl, 1.44 mM CaCl_2_, and 10 mM Hepes, pH 7.6) containing SUPERase·In RNase Inhibitor (40 U/ml). Cell viability following dissociation was evaluated through preliminary testing by diluting the cell suspension with a 1:1 erythrosin B solution (0.5 mg/ml). The dissociation protocol was optimized to ensure a cell mortality rate below 20%. Dissociated cells were immediately fixed in ice-cold ACME solution (*95*) containing SUPERase·In RNase Inhibitor (40 U/ml) for 30 min on ice under rotation. Fixed cells were kept in the ACME solution for up to a month at −20°C until further processing. To wash out the ACME fixative, 0.2 μm–filtered phosphate-buffered saline (PBS)/1% bovine serum albumin (BSA) containing SUPERase·In RNase Inhibitor (40 U/ml) was added 1:1 to the cell suspension and mixed gently by pipetting. Then, samples were centrifuged at 1000*g* for 10 min at 4°C using a swinging bucket centrifuge. The pellet was resuspended by adding 900 μl of 0.2 μm–filtered PBS/1% BSA containing SUPERase·In RNase Inhibitor (100 U/ml). To cryopreserve cells, 100 μl of dimethyl sulfoxide (DMSO) was added per tube and samples were stored at −80°C until further processing.

### Single-cell RNA sequencing

scRNA-seq for all samples was performed using 10x Genomics technology at the France Genomique functional genomics platform, IPMC, Nice-Sophia Antipolis. We conducted four capture runs for the *Clytia* samples, with one biological replicate per planula stage, each split into two technical replicates; and three capture runs each for the *Astroides* and *Pocillopora* samples, consisting of two biological replicates, one of which was split into two technical replicates.

Single-cell suspensions kept at −80°C were thawed on ice. To wash out the DMSO, cells were centrifuged twice at 1000*g* for 10 min at 4°C using a swinging bucket centrifuge. The supernatant was discarded, and the pellet was resuspended in 0.2 μm–filtered PBS/1% BSA containing SUPERase·In RNase Inhibitor (100 U/ml) after the first centrifugation and in 0.2 μm–filtered PBS/0.1% BSA containing SUPERase·In RNase Inhibitor (100 U/ml) after the second centrifugation. Cell concentration was roughly assessed using the Countess 3 Automated Cell Counter (Invitrogen). For one of the two *Astroides* biological replicates, cells were stained with 1/300 DRAQ5 (Thermo Fisher Scientific; stock, 5 mM) and sorted using a BD Influx cell sorter to discard debris and doublets. The other *Astroides* biological replicate and the *Clytia* and *Pocillopora* samples were processed directly after having optically examined the cell suspensions and discarded debris and cell clumps. Cells were loaded into the 10x Genomics platform for encapsulation with the capture goal between 7000 and 10,000 cells per sample. cDNA libraries were prepared according to the Chromium Next GEM Single Cell 3′ library preparation protocol version 3.1. Details of the four *Clytia*, four *Astroides*, and three *Pocillopora* libraries are provided in table S6. Sequencing was performed on an Illumina NextSeq2000 device with NextSeq 2000 P3 Reagents (100 cycles). Paired-end read lengths: read1 = 28 bp, Index1 = 10 bp, Index2 = 10 bp, and read2 = 90 bp (Index Kit TT SetA).

### Single-cell transcriptomic analysis

Individual 10× sample libraries were demultiplexed using Cell Ranger Makefastq version 7.0.0 with default settings. Reads for each sample were mapped using STARsolo (*96*) with the --soloCellFilter EmptyDrops_CR option and applying a 300 unique molecular identifier (UMI) cutoff using the genomes and gene models described above. Output count matrices were analyzed with Scanpy 1.9.1 (*97*). To exclude low-quality cells from downstream analysis, we filtered cells with fewer than 200 genes expressed and genes detected in fewer than three cells. In addition, we removed cells with high mitochondrial counts by setting thresholds for filtering based on visual examination of the percentage of mitochondrial counts across cells within each species’ dataset. *Clytia* cells were found to be more fragile upon dissociation. The percentages of mitochondrial counts were set to a maximum of 20% for *Clytia*, 0.4% for *Astroides*, and 5% for *Pocillopora*. Following this initial filtering, we ran each dataset through a standard Scanpy analysis, as follows, using default parameters. Counts per cell were normalized and log transformed, and highly variable genes were computed. Then, datasets were scaled and principal components (PCs) were computed on highly variable genes. Clustering was performed by first running the neighbors function using 50 neighbors, adjusting the number of PCs individually for each dataset (22 PCs for *Clytia*, 26 PCs for *Astroides*, and 23 PCs for *Pocillopora*), and then applying the Leiden graph-clustering algorithm (resolution of 1 for *Clytia* and *Pocillopora* and resolution of 0.9 *for Astroides*) (*48*). Cluster “marker genes” were identified by extracting significantly differentially expressed genes per cluster using the Scanpy’s rank_genes_groups function with the Wilcoxon rank-sum test method.

For the *Clytia* analysis, the Harmony algorithm (*47*) was used to correct the batch effects during the integration of datasets generated in this study with previously produced datasets for *Clytia* planula (*46*). For *Clytia* and *Astroides*, a cluster with no unique significantly represented genes when compared to other clusters and expression of marker genes across all clusters was removed from the datasets. The resulting datasets were reclustered and used for all downstream analyses.

### *A. calycularis* genome

Genomic DNA was extracted from the tissue of an isolated polyp of an adult colony. An individual polyp was separated from the colony by breaking its calcareous skeleton using pliers. The isolated polyp was snap frozen in liquid nitrogen and immediately thawed. Polyp tissue was then separated from the skeleton using an airbrush, collected in a tube, snap frozen in liquid nitrogen, and stored at −80°C until it was processed for DNA extraction. For DNA extraction, frozen polyp tissue was thawed on ice. Two ml of DNA extraction buffer [200 mM tris-HCl, pH 8.0, 20 mM EDTA, 0.1% SDS, 0.5 mg ml^−1^ proteinase K (Qiagen), and ribonuclease A (0.2 mg ml^−1^; Qiagen)] was added and incubated at 50°C for 2 hours until the solution became uniform and clear. An equal volume of phenol was added, mixed by inversion, and centrifuged for 30 min at 14,000 rpm (room temperature). Then, the upper layer was transferred to a clean tube and the same volume of chloroform was added. The solution was mixed by inversion and centrifuged for 30 min at 14,000 rpm (room temperature). A second chloroform wash was performed. The upper layer was transferred into a clean tube, and ×1/10 volume of 5 M NaCl and 0.7 volumes of isopropanol were added. The solution was gently mixed by pipetting, incubated for 30 min at room temperature, and centrifuged for 40 min at 14,000 rpm (room temperature). The DNA precipitate was rinsed with ice-cold 70% ethanol, dried, and dissolved into tris-EDTA buffer (10 mM tris-HCl, pH 8.0, and 1 mM EDTA, pH 8.0). A total of 30 μg of DNA was obtained from 28 mg of polyp tissue. In collaboration with M.-J. Arguel at the France Genomique platform, IPMC, Nice-Sophia Antipolis, longer DNA fragments were selected using The Short Reads Eliminator (SRE) Medium kit (Circulomics). Sequencing library preparation was performed at this platform using the Oxford Nanopore Ligation Sequencing Kit version14 (SQK-LSK114), and sequencing was done using a R10.4.1 PromethION flow cell.

Nanopore reads were assembled using Flye (2.9.1-b1780) (*98*) and https://github.com/fenderglass/Flye: “flye --nano-hq all.fastq.gz --scaffold --no-alt-contigs --out-dir scaff_flye --threads 64,” yielding a contig N50 of 205,099. We aligned all larval RNA-seq reads (101,361,743 100-bp paired-end reads) to this assembly using STAR (version 2.7.10b) (*93*). A total of 86% of reads was uniquely mapped. A sorted BAM file of these alignments was used as input to Stringtie (version 2.1.0) (*99*), with default parameters, to produce gene and transcript models. Protein sequences were predicted from these using Transdecoder (version 5.5.0), with the option to retain Pfam hits [http://transdecoder.github.io; (*100*)]. For further protein level analyses such as phylogeny inference, we retained the longest transcript per gene, giving 29,524 proteins. These data were 89% complete for BUSCO metazoa_odb9 (*101*).

### Orthology assignment and functional annotation

Orthologous pairs between all genes of our species of study were inferred from reciprocal best hits using the Smith-Waterman algorithm implemented in ssearch36 from the FASTA package (*102*). For selected genes, orthology assignments were refined by phylogenetic analysis. Tree inference (as opposed to pairwise orthologs) for particular protein families used the method outlined in (*103*). Alignments were constructed using programs from the HMMER3.3 package (http://hmmer.org/). Pfam hidden Markov models were searched against a protein database of representative metazoans (hmmsearch) using gathering threshold cutoffs (“--cut_ga”), and sequences were aligned (hmmalign) and then filtered and trimmed (esl-alimask “--pavg 0.5” and esl-alimanip “--minpp 0.3,” followed by “--lnfract 0.7”) on the basis of posterior probabilities of correct alignments and the length of the remaining sequence. Phylogenies were constructed using IQ-TREE, allowing the optimum model to be selected from the LG set (i.e., “-mset LG” in IQ-TREE).

For functional annotation, protein conserved domains were identified using hmmscan of the HMMER 3.3 package against the Pfam database (*104*). Analysis of GPCR and ion channels includes genes annotated with the following Pfam domains: 7tm_1, 7tm_2, and 7tm_3 (for GPCR); and Ion_trans, Lig_chan, Neur_chan_memb, and Neur_chan_LBD (for ion channels). Secreted proteins were annotated by the presence of a signal peptide using SignalP 5.0b (*105*).

### Protein 3D structure prediction

We used AlphaFold2 via ColabFold to predict the structure of PF1 (*37*, *38*). Structural novelty was assessed with Foldseek (*39*). We used boltz-1 (*64*) to predict the structure of putative cnidarian TauD with a bound ligand represented by SMILES strings.

### Fixation of specimens

For ISH, *Clytia* planulae were fixed in 3.7% formaldehyde/0.2% glutaraldehyde in PBS for 2 hours on ice, washed five times in PBST (PBS containing 0.1% Tween 20) for 10 min, then dehydrated in increasing concentrations of methanol in PBST (50, 75, and 100%), and stored at −20°C. For FISH, HCR, and immunostaining, *Clytia* planulae were fixed in a two-step incubation with (i) HEM buffer (0.1 M Hepes, pH 6.9, 50 mM EGTA, and 10 mM MgSO_4_) containing 3.7% formaldehyde and 0.2% glutaraldehyde for 5 min at room temperature and (ii) HEM buffer containing 3.7% formaldehyde for 2 hours at room temperature. After fixation, samples were washed four times for 5 min in PBST, dehydrated in increasing concentrations of methanol in PBST, and stored at −20°C in absolute methanol until use.

*Astroides* and *Pocillopora* planulae were first relaxed using 0.4 M menthol in 0.2-μm MFSW on ice for 5 min. Then, larvae were incubated for 5 min at room temperature and fixed for 1 hour in 3.7% formaldehyde in MFSW buffered to pH 8 with Hepes buffer (on ice). After four rinses with PBST, specimens were dehydrated through a graded series of methanol in PBST and stored at −20°C in absolute methanol until use.

### Whole-mount ISH, FISH, and in situ HCR

ISH was performed, as previously described (*36*), except that 4 M urea was used instead of 50% formamide in the hybridization buffer (*106*). ISH probes were synthesized from either pGEM-T Easy plasmids (following insert amplification by polymerase chain reaction) or expressed sequence tag clones (*107*).

FISH was performed for xloc_042849 and pp2 using DIG (digoxigenin)–labeled probes. Signal development was carried out with incubation overnight with a peroxidase-labeled anti-DIG antibody followed by washes in MABT (100 mM maleic acid, pH 7.5, 150 mM NaCl, and 0.1% Triton X-100). The fluorescence signal was developed using the TSA (Tyramide Signal Amplification) kit (TSA Plus Fluorescence Amplification kit, PerkinElmer, Waltham, MA) and Cy5 fluorophore (diluted 1/400 in TSA buffer: PBS/0.0015% H_2_O_2_) at room temperature for 30 min. The development reaction was stopped by replacing the solution with PBST. After three washes for 5 min in PBST, nuclei were stained using 1:2000 Hoechst dye 33258 (1 mg/ml stock; Sigma-Aldrich) in PBST overnight at 4°C.

HCRs were performed for *Clytia* and *Astroides* planulae in Eppendorf tubes using Bruce *et al.*’s protocol (*108*). Probes for HCR were designed using the probe generator program created by R. Null (https://github.com/rwnull/insitu_probe_generator). Between 12 and 38 probe pairs using the full coding sequence as a template were designed for each gene, ordered as oligo pools from IDT (Integrated DNA Technologies) and suspended in tris-EDTA buffer (10 mM tris, pH 8.0, and 0.1 mM EDTA) at 1 μM. Fixed specimens kept at −20°C were progressively rehydrated in PBST and permeabilized in detergent solution [1.0% SDS, 0.5% Tween 20, 150 mM NaCl, 1 mM EDTA (pH 8), and 50 mM tris-HCl at pH 7.5] for 1 hour. After four washes for 5 min in PBST at room temperature, the specimens were prehybridized in hybridization buffer (Molecular Instruments) for 1 hour at 37°C. The probes were then added to the hybridization buffer at a final concentration of 0.02 μM, and the samples were left to hybridize at 37°C overnight with gentle agitation. After hybridization, the samples were washed four times for 15 min in probe wash buffer (Molecular Instruments) at 37°C, followed by two washes in 5× saline sodium citrate buffer containing 0.1% Tween 20 (5× SSCT) for 5 min and two washes in 1× SSCT for 5 min at room temperature. They were then preamplified in amplification buffer (Molecular Instruments) for 30 min at room temperature. Meanwhile, H1 and H2 components of HCR hairpins B1 and B2 coupled to either Alexa 546 or Alexa 647 fluorophores (Molecular Instruments) were incubated separately at 95°C for 90 s, cooled down to room temperature in the dark, and then pooled together in the amplification buffer at a final concentration of 60 nM. The amplification was carried out overnight in the dark and at room temperature. Excess hairpins were removed through two 5-min washes and two 30-min washes in 1× SSCT, followed by two 5-min washes in PBST. Nuclei were stained using 1:2000 Hoechst dye 33258 (1 mg/ml stock; Sigma-Aldrich) in PBST overnight at 4°C.

### Immunostaining

Fixed specimens were gradually rehydrated from 100% methanol to PBST. *Clytia* larvae were extracted by performing two washes with PBS/0.2% Triton X-100 for 10 min on rotation. *Astroides* larvae were not extracted and instead were washed twice in PBST for 10 min on rotation. Samples were blocked in 0.2 μm–filtered blocking solution (3% BSA in PBST) for 1 hour at room temperature (on rotation). After blocking, they were incubated overnight at 4°C in the primary antibody solution. The primary antibodies used in this study were as follows: a mouse anti-LWamide antibody used at 1/20 [provided by O. Koizumi (*109*)]; a custom-made, affinity-purified rabbit polyclonal anti-QYFamide peptide antibody (produced by Covalab, Lyon) used at 13.5 ng/ml in blocking solution; and a custom-made, affinity-purified rat polyclonal anti-GLWamide antibody (Covalab) used at 2.45 ng/ml in blocking solution. Following primary antibody incubation, samples were washed five times for 30 min each in PBST and incubated with fluorophore-conjugated secondary antibodies diluted 1:200 [anti-mouse immunoglobulin (Ig), anti-rat Ig, and anti-rabbit Ig; the Jackson Laboratory] and Hoechst dye 33342 1:2000 in PBST overnight at 4°C. Samples were then washed twice in PBST for 10 min and twice in 1× PBS for 5 min before mounting.

### Microscopy

Colorimetric ISH samples were mounted in 50% glycerol and imaged using a Zeiss Axio Imager 2 microscope. Images were processed with ImageJ version 2.3.0/1.53f (*110*). For fluorescently labeled samples (FISH, HCR, and immunohistochemistry), *Clytia* samples were mounted in Citifluor antifade mountant (Citifluor-EMS) and *Astroides* samples were dehydrated through a glycerol series and mounted in 90% glycerol/1× PBS. Images were acquired using Leica Stellaris 5 or Sp8 laser scanning confocal microscopes, and *Z*-stack maximum intensity projections were generated using ImageJ software.

### Settlement assays

To test the effect of different molecules on *Clytia* settlement, four-well 1-ml plastic culture dishes (Thermo Fisher Scientific) were conditioned to generate a settlement-inducing biofilm. Culture dishes were left incubating in a sea urchin tank of the IMEV/EMBRC-Fr Aquarium Service with a continuous flow of natural seawater containing different types of algae and microorganisms for a week. After this time period, a biofilm was generated on the surfaces of the wells, which efficiently induces *Clytia* planula settlement after overnight incubation (>90% of settlement). Treatments were performed in conditioned culture wells with each well containing 100 or 50 late 2-day planulae (52 hpf). Taurine, glutamate, betaine, GABA, baclofen, and homotaurine (Sigma-Aldrich) were dissolved in nonfiltered ASW at concentrations of 10^−3^, 10^−4^, and 10^−5^ M diluted from 10^−2^ M stocks in H_2_O just before use. Each replicate contained a control condition with larvae incubated in nonfiltered ASW. Settlement was scored about 16 to 20 hours after the start of the incubation. For *Astroides*, incubations were in 24-well plastic dishes containing five planulae per well. Settlement was scored after 1 week. Taurine and glutamate were dissolved in natural seawater at concentrations of 10^−3^, 10^−4^, 10^−5^, and 10^−6^ M. Each replicate included a control condition with larvae incubated in natural seawater only. Solutions were replaced daily to compensate for molecule degradation. To assess the rate of settlement, settled larvae were counted daily.

## Supplementary Materials

### The PDF file includes:

Figs. S1 to S17 Legends for tables S1 to S6 References
