# Supplementary material for: Aboral cell types of Clytia and coral larvae have shared features and link taurine to the regulation of settlement
Source: Sci Adv. 2025 May 16;11(20):eadv1159. doi: 10.1126/sciadv.adv1159 (PMC12225738; doi:10.1126/sciadv.adv1159)
Supplement: Supplementary file 1 — Figs. S1 to S17 Legends for tables S1 to S6 References [file sciadv.adv1159_sm.pdf]

Science Advances
AAAS

Supplementary Materials for

Aboral cell types of *Clytia* and coral larvae have shared features and link taurine to the regulation of settlement

Julia Ramon-Mateu *et al.*

Corresponding author: Evelyn Houliston, evelyn.houliston@imev-mer.fr;
Richard R. Copley, richard.copley@imev-mer.fr

Sci. Adv. **11**, eadv1159 (2025)
DOI: 10.1126/sciadv.adv1159

The PDF file includes:

Figs. S1 to S17
Legends for tables S1 to S6
References

Other Supplementary Material for this manuscript includes the following:

Tables S1 to S6

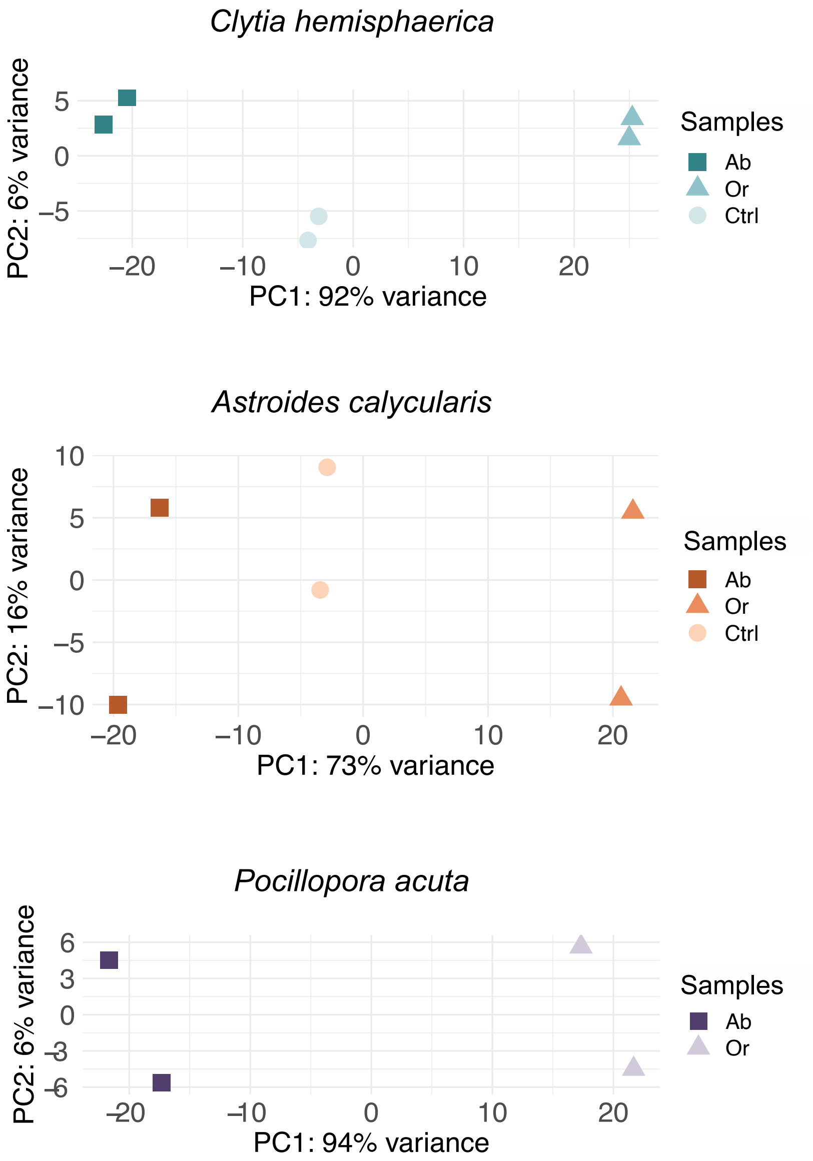

Fig. S1. Principal Component Analysis (PCA) of planula Aboral/Oral transcriptomes. PCA plots of RNA-seq libraries for each species generated using normalized counts (see Methods). Samples for *Clytia* (top) and *Astroides* (middle) are aboral (Ab), oral (Or), and uncut control (Ctrl); for *Pocillopora* (bottom), the samples are aboral (Ab) and oral (Or).

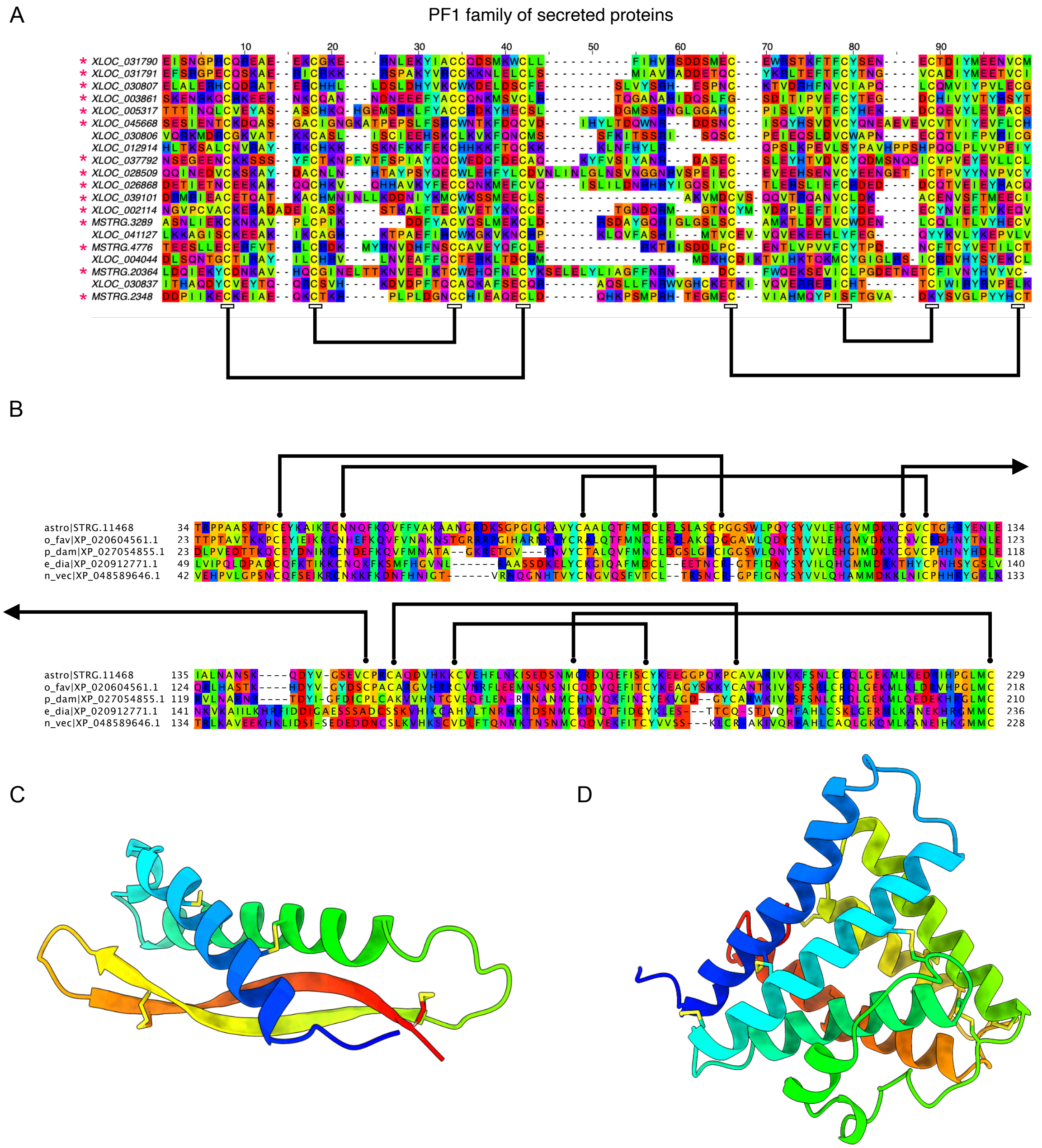

Fig. S2. Aboral-enriched short secreted proteins with similar features. (A and B) Protein sequence alignments of the *Clytia* PF1 family of secreted proteins (A) and an anthozoan-specific secreted protein (B). Alignments have been trimmed to the conserved cores. Cysteine residues are colored yellow. Potential disulfide bridges, inferred from predicted 3D structures, are indicated with black bracket lines. Red asterisks mark PF1 members enriched at the aboral end. (C and D) Predicted three-dimensional structures of PF1 secreted proteins and the anthozoan secreted protein, respectively. The N-terminus is colored blue, and the C-terminus is red. Disulfide bridges are shown as sticks, with cysteines in yellow.

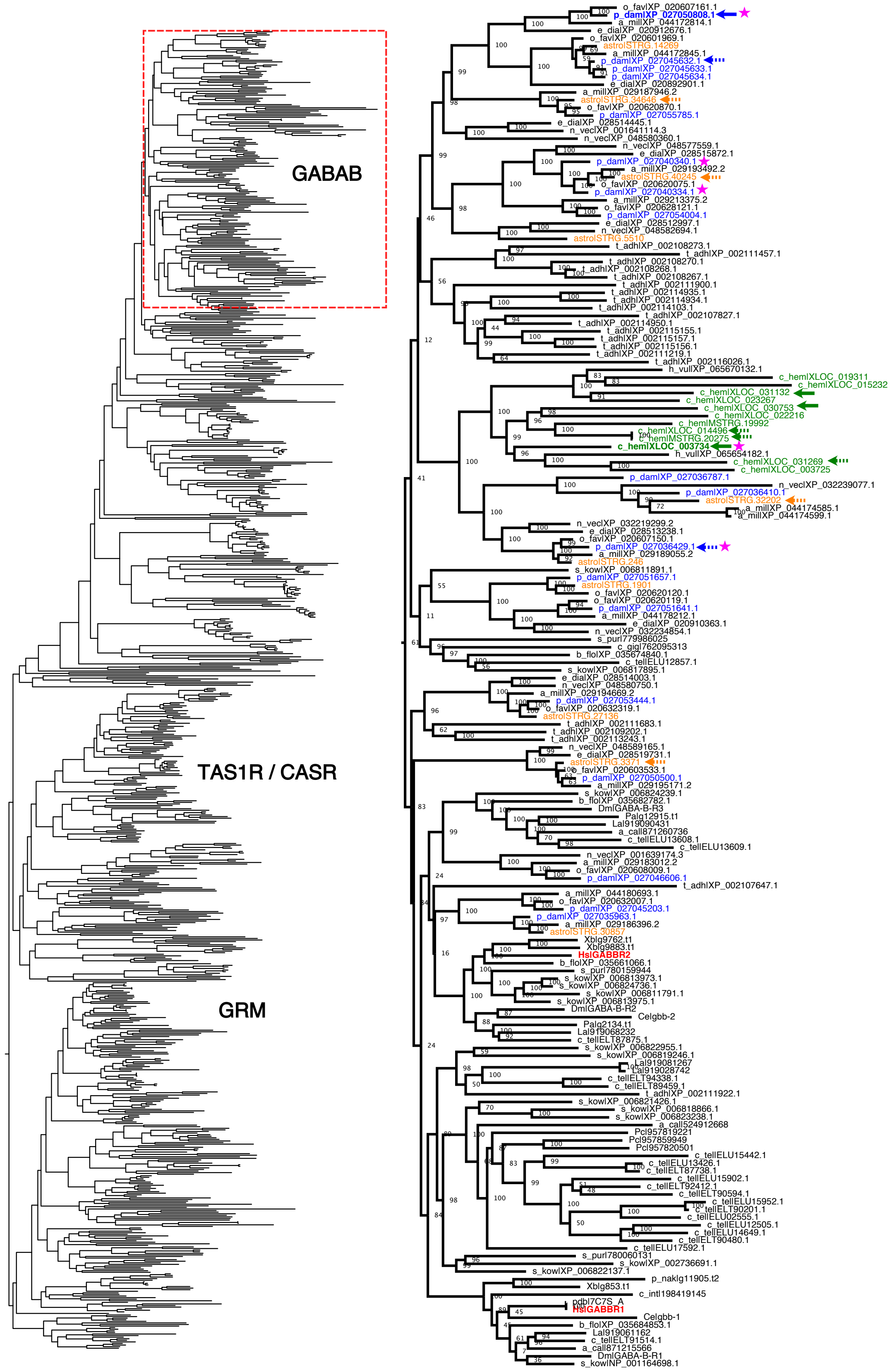
GABAB
TAS1R / CASR
GRM
HsGABBR2
HsGABBR1

Fig. S3. Maximum Likelihood phylogenetic analysis of metabotropic-type GPCRs. The tree on the left includes putative metabotropic receptor sequences categorized by including the Pfam domains ANF_receptor and 7tm_3. GABAB: Metabotropic GABA receptor; TAS1R/CASR: Taste Receptors type 1/Calcium-Sensing Receptors; GRM: Metabotropic Glutamate receptors. The tree on the right is a zoomed view of the region outlined by the dotted red box. Values on nodes = Ultrafast bootstrap values from IQ-TREE (1000 replicates). Sequences from *Clytia*, *Astroides*, and *Pocillopora* are highlighted in green, orange, and blue, respectively, and human sequences are marked in red. Arrows indicate aboral-enriched sequences (continuous lines lfc>1, dotted lines lfc>0). Magenta stars indicate expression in GLWamide cells. p_dam = *Pocillopora damicornis*; o_fav = *Orbicella faveolata*; astro = *Astroides calycularis*; a_mil = *Acropora millepora*; e_dia = *Exaiptasia diaphana*; n_vec = *Nematostella vectensis*; t_adh = *Trichoplax adhaerens*; s_kow = *Saccoglossus kowalevskii*; Dm = *Drosophila melanogaster*; a_cal = *Aplysia californica*; c_tel = *Capitella teleta*; La = *Lingula anatina*; Xb = *Xenoturbella bockii*; p_nak = *Praesagittifera naikaiensis*; b_flo = *Branchiostoma floridae*; Hs = *Homo sapiens*; pdb = Protein Data Bank (3D structures); c_int = *Ciona intestinalis*; Ce = *Caenorhabditis elegans*; s_pur = *Strongylocentrotus purpuratus*; Pc = *Priapulus caudatus*; Pa = *Phoronis australis*; c_hem = *Clytia hemisphaerica*; h_vul = *Hydra vulgaris*.

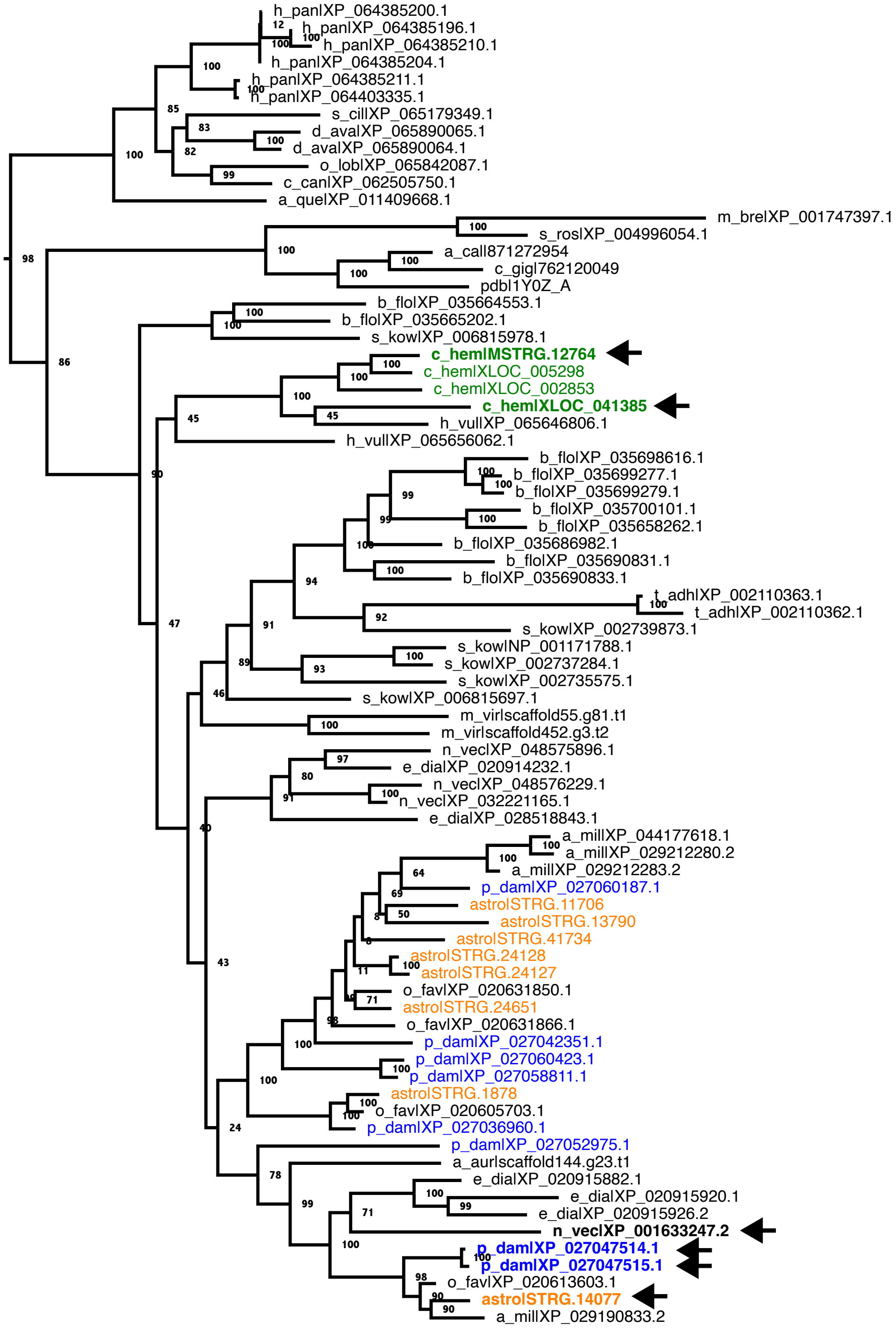

Fig. S4. Maximum Likelihood phylogenetic analysis of TauD proteins. A robust subclade of the overall TauD phylogeny is shown. Sequences from *Clytia*, *Astroides*, and *Pocillopora* are highlighted in green, orange, and blue, respectively. Black arrows indicate expression enriched in the aboral end. The *Nematostella* sequence (in bold black) is equivalent to the one in (*33*). Values on nodes = Ultrafast bootstrap values from IQ-TREE (1000 replicates). h_pan = *Halichondria panicea*; s_cil = *Sycon ciliatum*;

d_ava = *Dysiea avara;* o_lob = *Oscarella lobularis*; c_can =*Corticium candelabrum*; a_que = *Amphimedon queenslandica*; m_bre = *Monosiga brevicollis*; s_ros = *Salpingoeca rosetta*; a_cal = *Aplysia californica*; c_gig = Crassostrea gigas; pdb = Protein Data Bank (3D structures); b_flo = *Branchiostoma floridae*; s_kow = *Saccoglossus kowalevskii*; c_hem = *Clytia hemisphaerica*; h_vul = *Hydra vulgaris*; t_adh = *Trichoplax adhaerens*; m_vir = *Morbakka virulenta*; N_vec = *Nematostella vectensis;* e_dial = *Exaiptasia diaphana*; p_dam = *Pocillopora damicornis*; a_mil = *Acropora millepora*; astro = *Astroides calycularis*; o_fav = *Orbicella faveolata*; a_aur = *Aurelia aurita*.

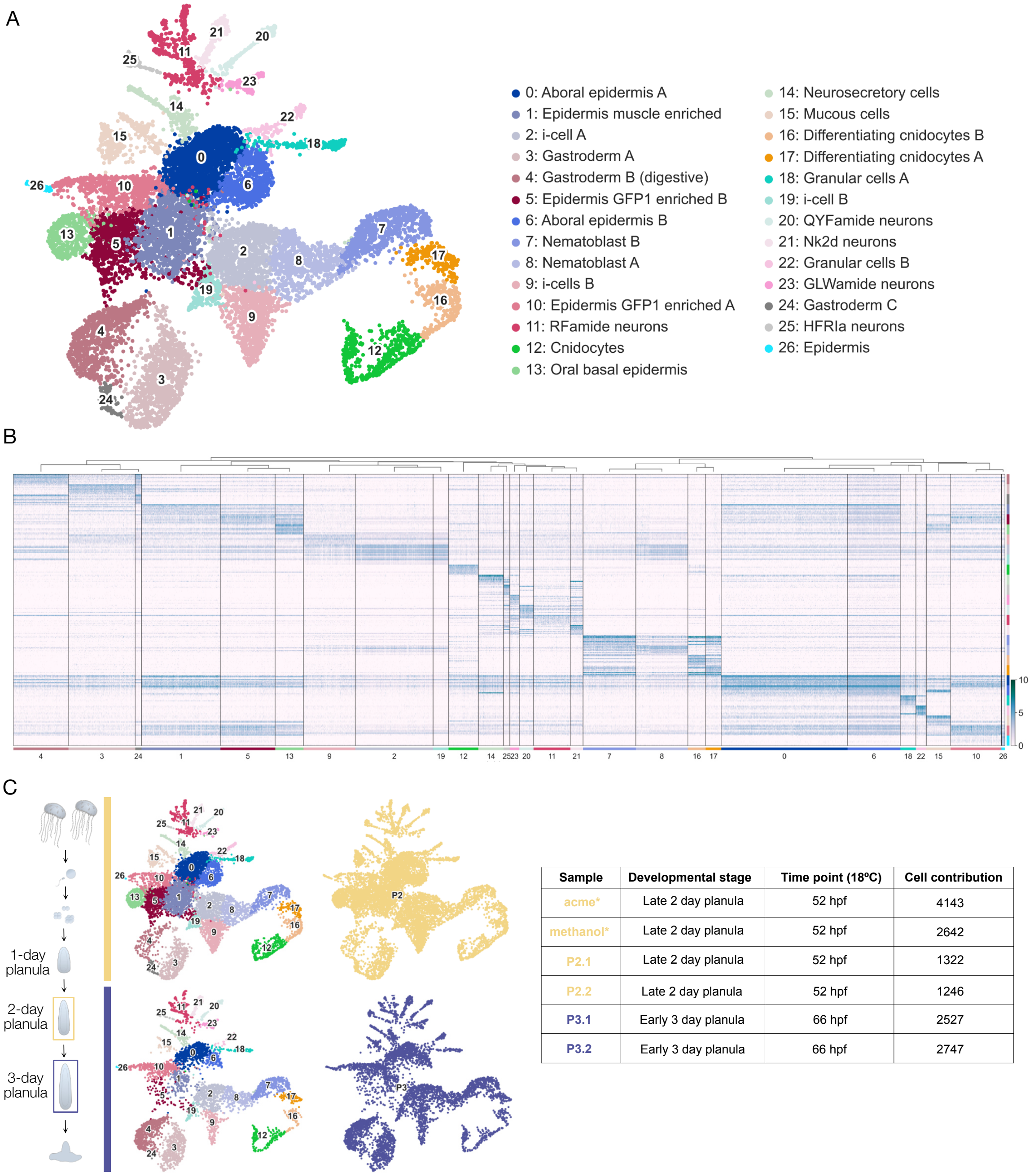

Sample	Developmental stage	Time point (18ºC)	Cell contribution
acme*	Late 2 day planula	52 hpf	4143
methanol*	Late 2 day planula	52 hpf	2642
P2.1	Late 2 day planula	52 hpf	1322
P2.2	Late 2 day planula	52 hpf	1246
P3.1	Early 3 day planula	66 hpf	2527
P3.2	Early 3 day planula	66 hpf	2747

Fig. S5. Cluster annotation and sample contribution for the *Clytia* planula scRNA-seq dataset. (A) UMAP representation of *Clytia* planula scRNA-seq data with all 27 clusters labeled by cell type identity. (B) Heatmap of the 10 top marker genes for each cluster. Cluster color labels correspond to (A). (C) On the left, UMAP representation of cells sampled from 2-day (top) and 3-day planulae (bottom). Left plots are labeled by cell cluster and right plots are labeled by developmental stage (color code matching to the schematics on the left). On the right, a table with information for each library contributing to the dataset.

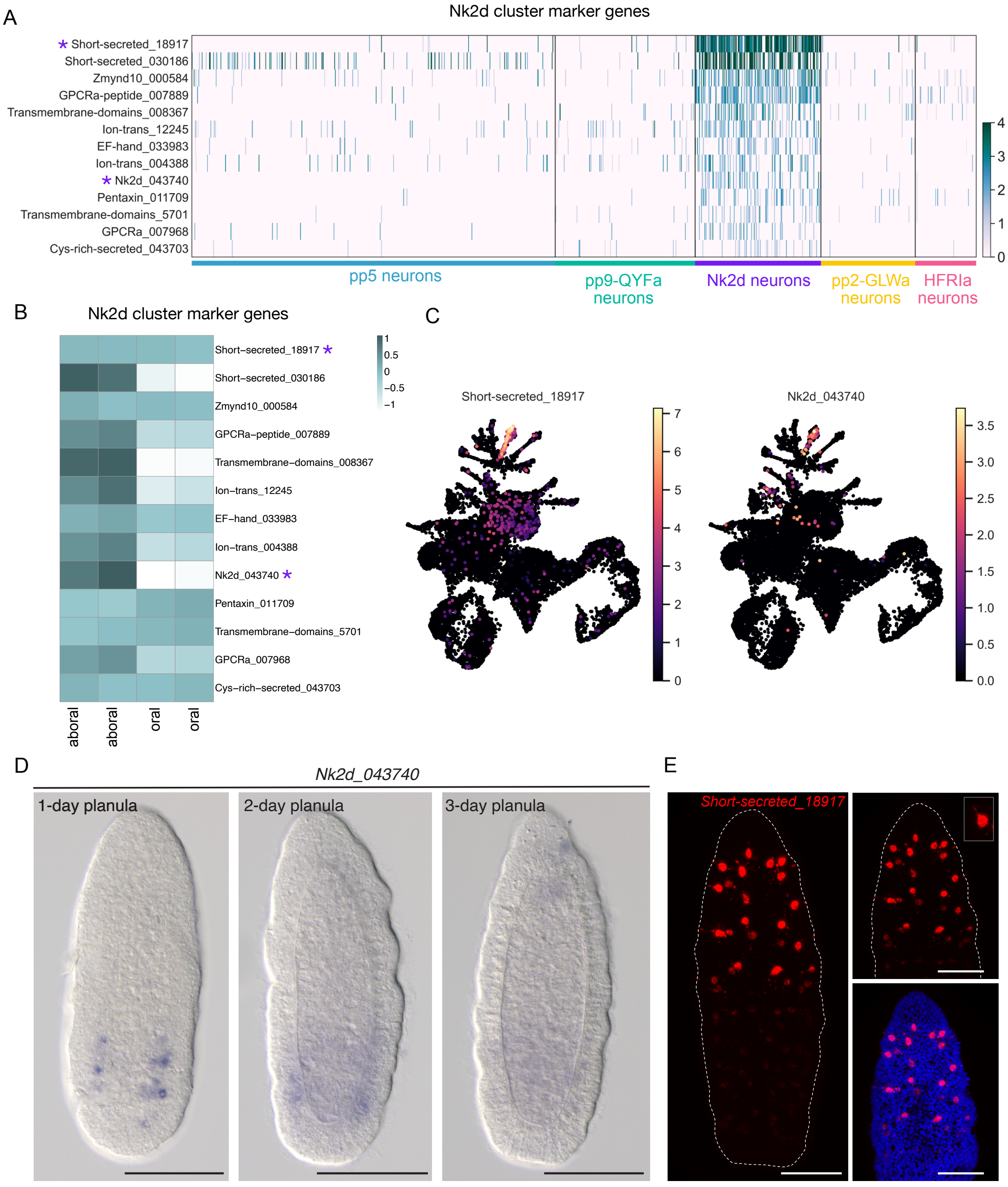

Fig. S6. Characterization of the "Nk2d neural" cluster. (A) Heatmap of the marker genes for the Nk2d cell cluster. Purple asterisks indicate the genes shown in (C, D, E). Only neural clusters are included. Cell cluster annotation labels correspond to Fig. 2C and Fig. S4. (B) Heatmap of aboral/oral expression (normalized counts) for the genes shown in (A). The "Nk2d cell" cluster marker genes included a mixture of aboral-enriched genes and genes with no aboral or oral enrichment. (C) UMAP plots of an aboral

enriched marker gene (Nk2d_043740) and a homogeneously expressed marker gene (Short-secreted_18917). Expression of these two genes maps to subpopulations within the cluster. (D) ISH for Nk2d_043740 in 1-day, 2-day planula and 3-day planula stages. Expression is detected at the 1-day stage in cells at the base of the epidermis at the aboral end. Fainter signal was seen in the same area at 2-day planula. (E) HCR for Short-secreted_18917 in a 2-day planula showing expression in ganglionic-like neurons brightly stained in the oral half. Panels on the right show a magnification of the oral end. Inset shows close-up of an individual cell body. Short-secreted_18917 (red) and Hoechst nuclear stain (blue). Planulae all oriented with the oral pole uppermost. Scale bars, 100 μm.

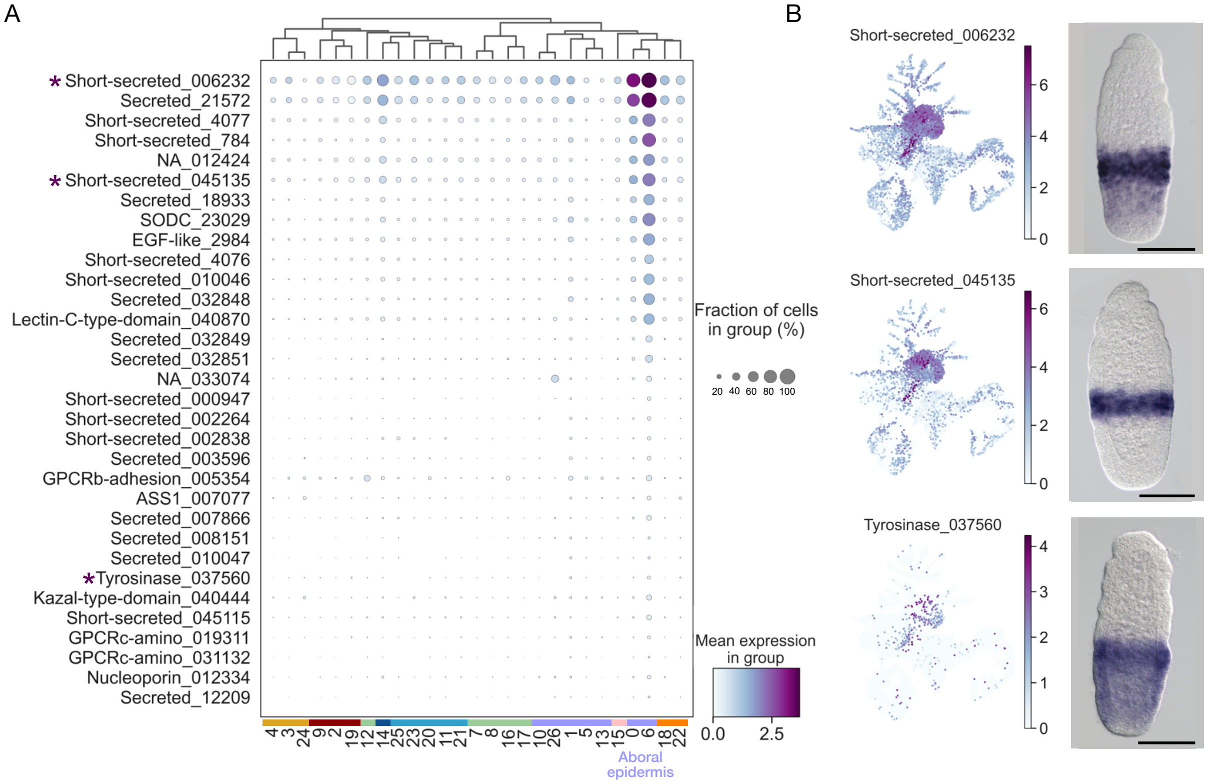

Fig. S7. Putative aboral epidermis clusters in the *Clytia* planula cell atlas. (A) Gene expression dot plot of specific markers for the epidermal clusters 0 and 6. Gene IDs are listed on the left and cluster identities are indicated at the bottom. The color coding for the clusters corresponds to the cell class annotations in Fig. 2. The color gradient indicates average expression, and the size of the dots indicates the percentage of cells expressing the gene. Purple asterisks indicate the genes shown in (B). Note that the cluster markers include several short secreted proteins, a putative neuropeptide (Che-pp6 which is predicted to generate SRLLFamide peptides) and two class C GPCRs. (B) UMAP plots for three markers and corresponding in situ hybridization patterns in 2-day planula stage. Scale bars, 100 μm.

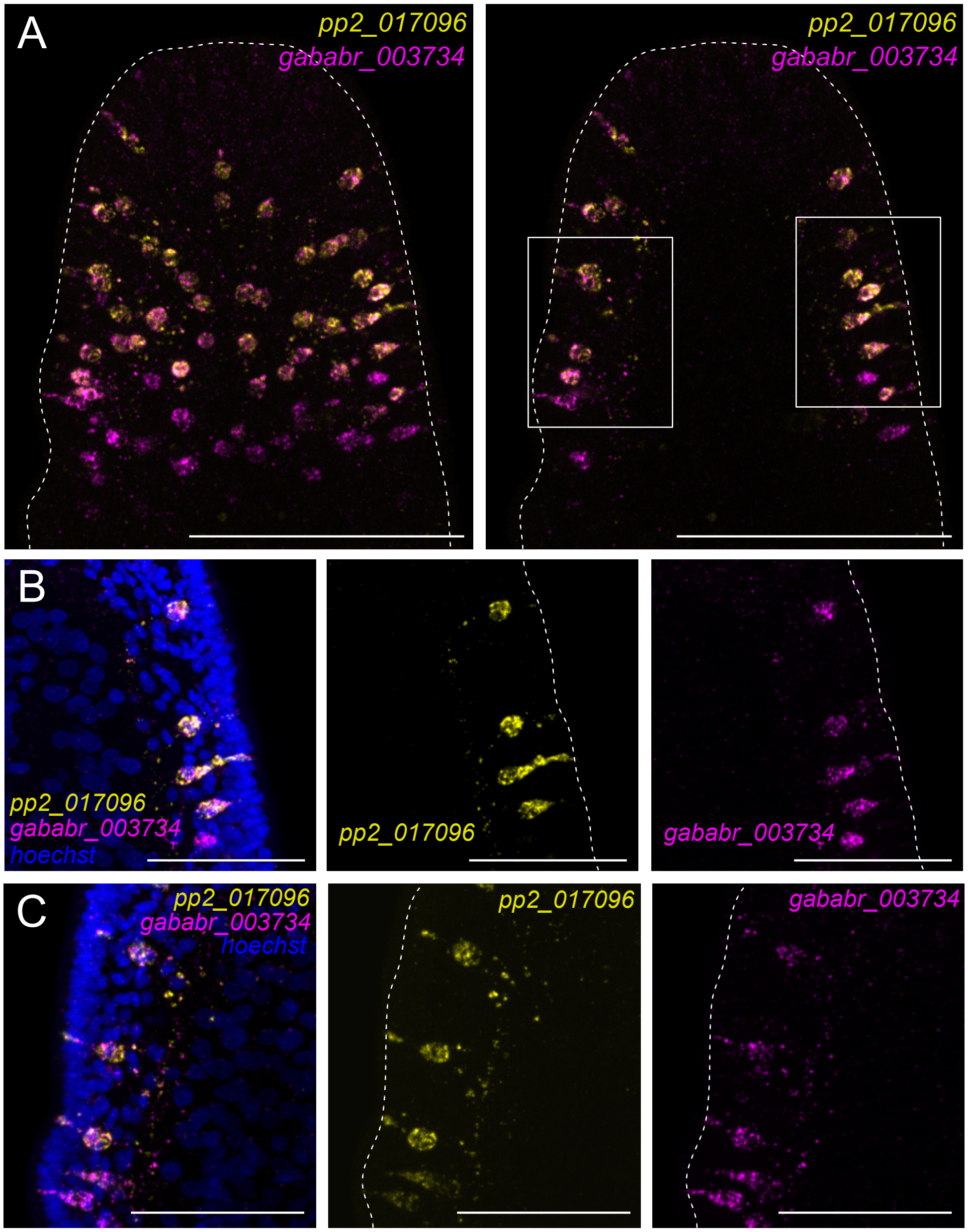

Fig. S8. Co-expression of the GLWamide precursor pp2 and GABA$_B$R-like in aboral cells of the *Clytia* planula. Double FISH showing co-expression of pp2 (yellow) and GABA$_B$R (magenta) mRNAs in the same cells of the aboral epidermis. (A) Maximum intensity projections of confocal Z-stacks of the aboral pole. Surface and medial stacks are shown on the left; selected medial stacks are shown on the right. Scale bars, 100 μm. (B and C) Maximum intensity projections of confocal Z-stacks of outlined regions in (A): (B) corresponds to the right square and (C) to the left. pp2 (yellow), GABA$_B$R (magenta), and Hoechst nuclear stain (blue). Single channels are shown in the middle and right panels. Scale bars, 50 μm.

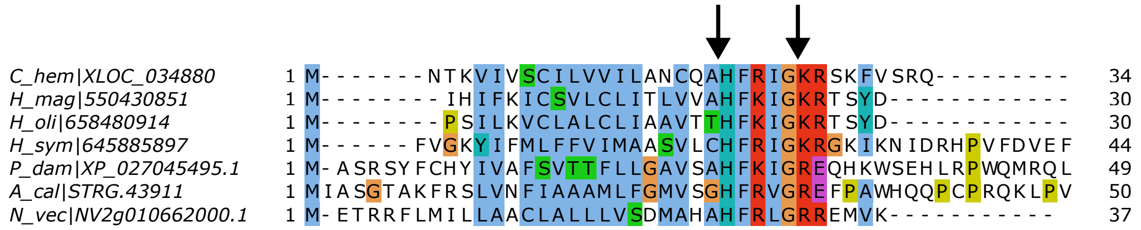

Fig. S9. Putative HFRIamide neuropeptide in Cnidaria. Alignment of the N-termini of HFRIamide protein precursors from selected of hydrozoan and anthozoan species. The arrows mark the likely signal peptide cleavage site (left) and neuropeptide cleavage site (right). C_hem = *Clytia hemisphaerica*; H_mag = *Hydra magnipapillata*; H_oli = *Hydra oligactis*; H_sym = *Hydractinia symbiolongicapus*; P_dam = *Pocillopora damicornis*; A_cal = *Astroides calycularis*; N_vec = *Nematostella vectensis*. Coloring conventions are those of Clustal (*114*).

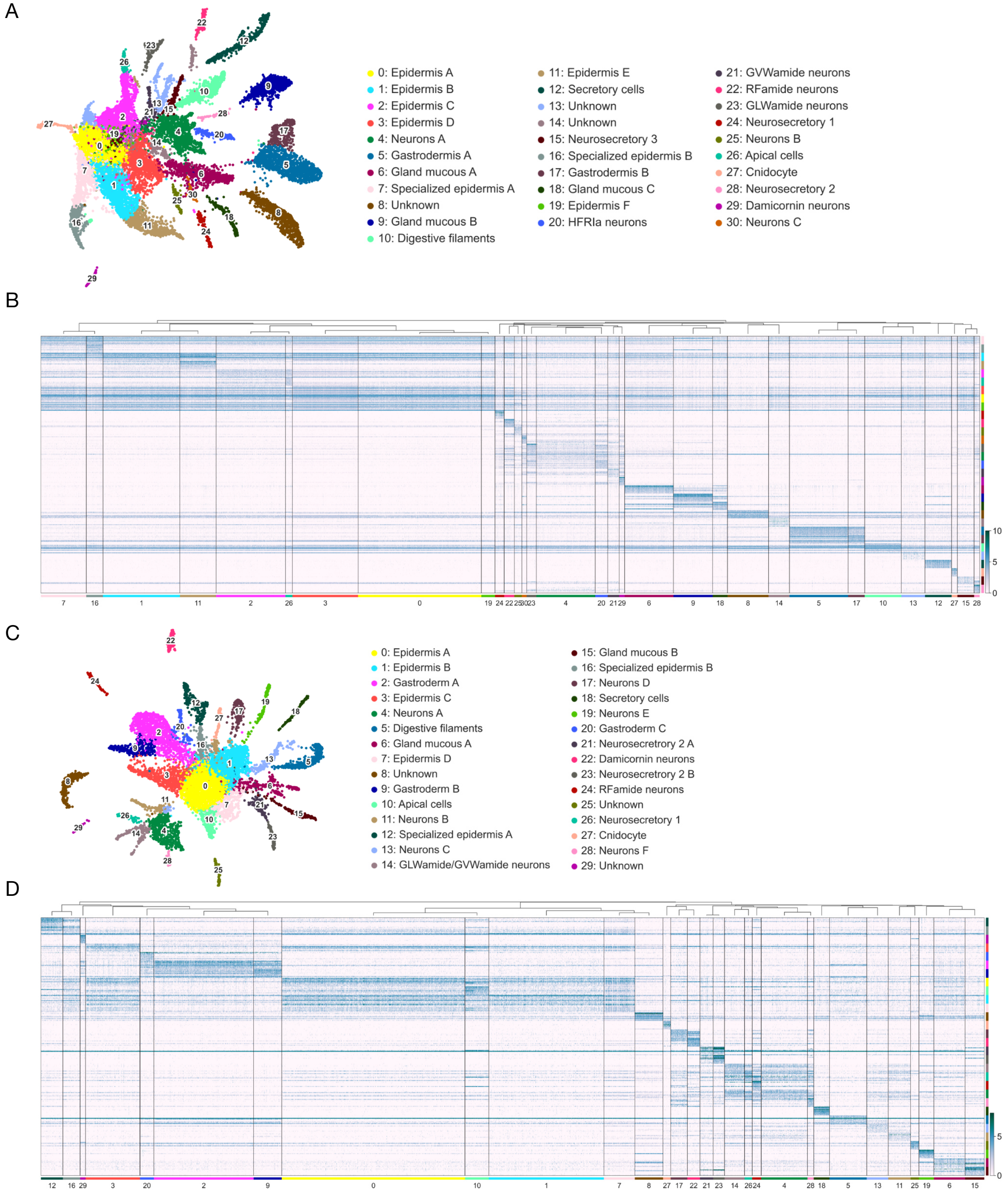

Fig. S10. The *Astroides* and *Pocillopora* planula cell atlases. (A and C) UMAP representations of scRNA-seq data from *Astroides* (A) and *Pocillopora* (C) planulae, with all clusters labeled by cell type identity. (B and D) Heatmaps of the 10 top marker genes for each cluster in *Astroides* and *Pocillopora*, respectively. The cluster color labels correspond to their respective atlases.

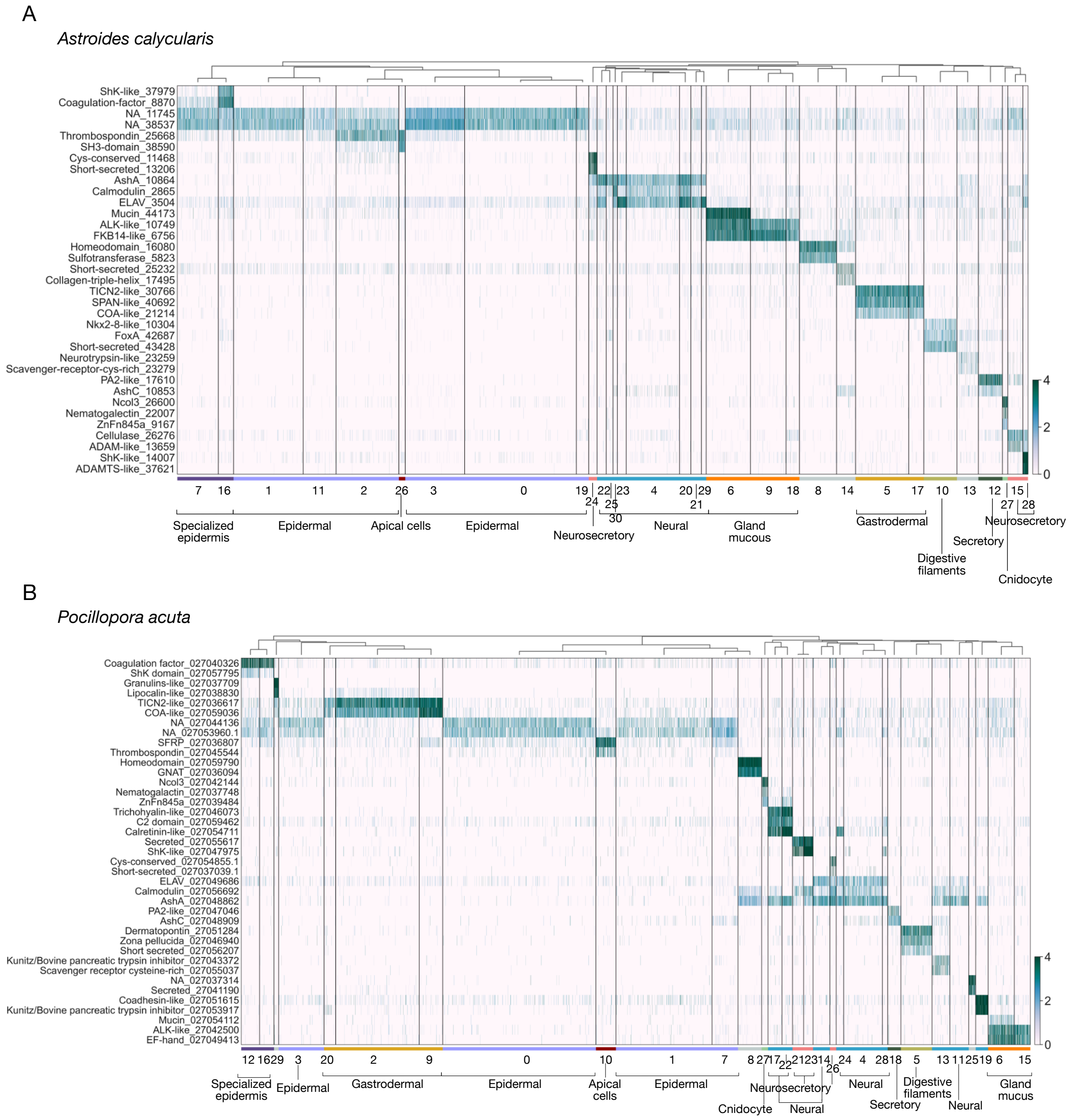

Fig. S11. Annotation of coral planula cell atlases. Heatmap of diagnostic marker genes used to annotate cell type classes for *Astroides* (top) and *Pocillopora* (bottom) planula scRNA-seq clusters. Gene names are taken from orthology assignments. In cases where assignment was not clear, the conserved protein domain is indicated. NA indicates absence of orthology assignment and conserved domains. Trailing numbers are unique gene identifiers from this project.

GENERAL IDENTITY	CLUSTER		CELL TYPE	DIAGNOSTIC MARKERS	REFERENCE
	A. calycularis	*P. acuta*			
Epidermal	0, 1, 2, 3, 11, 19	0, 1, 3, 7		Sox3: STRG.11680 / XP_027038477.1	*N. vectensis:* Steger et al. (ref 61)
				STRG.11745 / XP_027044136.1	
				STRG.38537 / XP_027053960.1	
Spezialized epidermis	7, 16	12, 16		Shk domain-like: STRG.37979 / XP_027057795.1	
				STRG.8870 / XP_027040326.1	
Apical cells	26	10		ISX-like: STRG.15235 / XP_027046639.1	*N. vectensis*: Gilbert et al. (ref 6) & Sabin et al. (ref 7)
				FoxQ2a: STRG.21723 / XP_027053483.1	*N. vectensis*: Sinigaglia et al. (ref 20)
				FGF: STRG.38785 / XP_027049367.1	*N. vectensis*: Rentzsch et al. (ref 31)
				PoxA: STRG.27319 / XP_027045474.1	*N. vectensis*: Gilbert et al. (ref 6)
Gastroderm	5, 17	2, 9, 20		ZF-C3H11: STRG.10901 / XP_027055412.1	*N. vectensis:* Steger et al. (ref 61)
				Testican2-like: STRG.30766 / XP_027036617.1	*N. vectensis:* Steger et al. (ref 61)
				COA-like2: STRG.21214 / XP_027059036.1	*N. vectensis:* Steger et al. (ref 61)
Digestive filaments	10	5		Nkx2-8-like: STRG.10304 / XP_027038915.1	*S. pistillata*: Levy et al. (ref 59)
				OIT3-like12: STRG.21702 / XP_027046940.1	*S. pistillata*: Levy et al. (ref 59)
Neural	4, 20, 21, 22, 23, 25, 30, 29	4, 11, 13,14, 17, 19, 22, 24, 28		AshA: STRG.10864 / XP_027048862.1	*N. vectensis*: Layden et al. (ref 62) & Steger et al. (ref 61)
				ELAV - STRG.3504 / XP_027049686.1	
	21	14	GVWamide neurons	GVWamide: STRG.21557 / XP_027059473.1	
	22	24	RFamide neurons	RFamide: STRG.11240 / XP_027051563.1	
	23	14	GLWamide neurons	GLWamide: STRG.15971 / XP_027060553.1	
	29	22	Damicornin neurons	Damicornin: STRG.4391 / XP_027051004.1	
	20	-	HFRIa neurons	HFRIa: STRG.43911 / XP_027045495.1	
Gland mucous	6, 9, 18	6, 15		RFX4: STRG.29170 / XP_027047616.1	*N. vectensis:* Steger et al. (ref 61) & Gilbert et al. (ref 6)
				Mucin : STRG.44173 / XP_027054112.1	*N. vectensis:* Steger et al. (ref 61)
				ALK-like: STRG.10749 / XP_027042500.1	*N. vectensis:* Steger et al. (ref 61)
Neurosecretory	15, 24, 28	21, 23, 26			
	24	26	Neurosecretory 1	Secreted, Cys-conserved: STRG.11468 / XP_027054855.1	
				Nkx2.5: STRG.11493 / XP_027041575.1	
	28	21, 23	Neurosecretory 2	Shk domain-like: STRG.14007 / XP_027047975.1	
				Pax6: STRG.28117 / XP_027036201.1	
Secretory	12	18		AshC: STRG.10853 / XP_027048909.1	*N. vectensis:* Steger et al. (ref 61)
				PA2-like2: STRG.17610 / XP_027047046.1	
Cnidocyte	27	27		Znfn845: STRG.9167 / XP_027039484.1	*C.hemisphaerica:* Chari et al. (ref 57) *N. vectensis*: Babonis et al. (ref 115)
				Ncol3: STRG.26600 / XP_027042144.1	*C.hemisphaerica:* Denker et al. (ref 116) *N. vectensis:* Sunagar et al. (ref 117)
				Nematogalectin-like: STRG.22007 / XP_027037748.1	*N. vectensis:* Babonis & Martindale (ref 118)

Fig. S12. Diagnostic marker genes used for assigning cell type identities in the coral planula atlases.

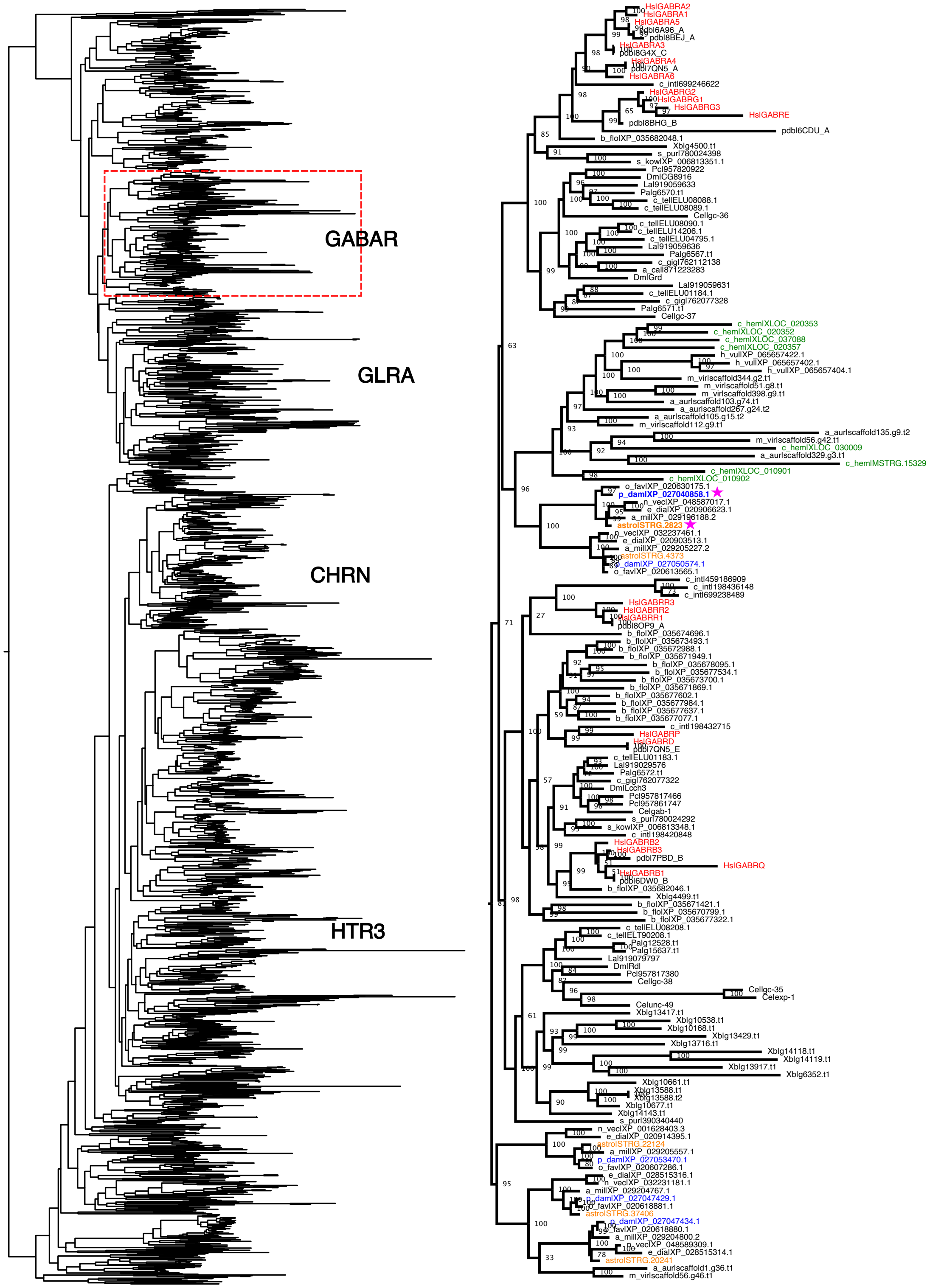

GABAR
GLRA
CHRN
HTR3
HsIGABRA2
HsIGABRA1
HsIGABRA5
pdbl6A96_A
pdbl8BEJ_A
HsIGABRA3
pdbl8G4X_C
HsIGABRA4
pdbl7QN5_A
HsIGABRA6
c_intl699246622
HsIGABRG2
HsIGABRG1
HsIGABRG3
HsIGABRE
pdbl8BHG_B
pdbl6CDU_A
b_flolXP_035682048.1
Xblg4500.t1
s_purl780024398
s_kowlXP_006813351.1
Pcl957820922
DmlCG8916
Lal919059633
Palg6570.t1
c_tellELU08088.1
c_tellELU08089.1
Cellgc-36
c_tellELU08090.1
c_tellELU14206.1
c_tellELU04795.1
Lal919059636
Palg6567.t1
c_gigl762112138
a_call871223283
DmlGrd
Lal919059631
c_tellELU01184.1
c_gigl762077328
Palg6571.t1
Cellgc-37
c_hemlXLOC_020353
c_hemlXLOC_020352
c_hemlXLOC_037088
c_hemlXLOC_020357
h_vullXP_065657422.1
h_vullXP_065657402.1
h_vullXP_065657404.1
m_virlscaffold344.g2.t1
m_virlscaffold51.g8.t1
m_virlscaffold398.g9.t1
a_aurlscaffold103.g74.t1
a_aurlscaffold267.g24.t2
a_aurlscaffold105.g15.t2
m_virlscaffold112.g9.t1
a_aurlscaffold135.g9.t2
m_virlscaffold56.g42.t1
c_hemlXLOC_030009
a_aurlscaffold329.g3.t1
c_hemlMSTRG.15329
c_hemlXLOC_010901
c_hemlXLOC_010902
o_favlXP_020630175.1
p_damlXP_027040858.1
n_veclXP_048587017.1
e_dialXP_020906623.1
a_millXP_029196188.2
astrolSTRG.2823
n_veclXP_032237461.1
e_dialXP_020903513.1
a_millXP_029205227.2
astrolSTRG.4373
p_damlXP_027050574.1
o_favlXP_020613565.1
c_intl459186909
c_intl198436148
c_intl699238489
HsIGABRR3
HsIGABRR2
HsIGABRR1
pdbl8OP9_A
b_flolXP_035674696.1
b_flolXP_035673493.1
b_flolXP_035672988.1
b_flolXP_035671949.1
b_flolXP_035678095.1
b_flolXP_035677534.1
b_flolXP_035673700.1
b_flolXP_035671869.1
b_flolXP_035677602.1
b_flolXP_035677984.1
b_flolXP_035677637.1
b_flolXP_035677077.1
c_intl198432715
HsIGABRP
HsIGABRD
pdbl7QN5_E
c_tellELU01183.1
Lal919029576
Palg6572.t1
c_gigl762077322
DmlLcch3
Pcl957817466
Pcl957861747
Celgab-1
s_purl780024292
s_kowlXP_006813348.1
c_intl198420848
HsIGABRB2
HsIGABRB3
pdbl7PBD_B
HsIGABRQ
HsIGABRB1
pdbl6DW0_B
b_flolXP_035682046.1
Xblg4499.t1
b_flolXP_035671421.1
b_flolXP_035670799.1
b_flolXP_035677322.1
c_tellELU08208.1
c_tellELT90208.1
Palg12528.t1
Palg15637.t1
Lal919079797
DmlRdl
Pcl957817380
Cellgc-38
Cellgc-35
Celexp-1
Celunc-49
Xblg13417.t1
Xblg10538.t1
Xblg10168.t1
Xblg13429.t1
Xblg13716.t1
Xblg14118.t1
Xblg14119.t1
Xblg13917.t1
Xblg6352.t1
Xblg10661.t1
Xblg13588.t1
Xblg13588.t2
Xblg10677.t1
Xblg14143.t1
s_purl390340440
n_veclXP_001628403.3
e_dialXP_020914395.1
astrolSTRG.22124
a_millXP_029205557.1
p_damlXP_027053470.1
o_favlXP_020607286.1
e_dialXP_028515316.1
n_veclXP_032231181.1
a_millXP_029204767.1
p_damlXP_027047429.1
o_favlXP_020618881.1
astrolSTRG.37406
p_damlXP_027047434.1
o_favlXP_020618880.1
a_millXP_029204800.2
n_veclXP_048589309.1
e_dialXP_028515314.1
astrolSTRG.20241
a_aurlscaffold1.g36.t1
m_virlscaffold56.g46.t1

Fig. S13. Maximum Likelihood phylogenetic analysis of ligand-gated ion channels. The tree on the left includes putative ligand-gated ion channel sequences categorized by including the Pfam domains Neur_chan_LBD and Neur_chan_memb. GABAR: ionotropic GABA receptors; GLRA: Glycine receptors; CHRN = Acetylcholine receptors; HTR3: 5-hydroxytryptamine receptors 3. The tree on the right is a zoomed view of the region outlined by the dotted red box. Values on nodes = Ultrafast bootstrap values from IQ-TREE (1000 replicates). Sequences from *Clytia*, *Astroides*, and *Pocillopora* are highlighted in green, orange, and blue, respectively, and human sequences are marked in red. Magenta stars indicate expression in GLWamide cells. Hs = *Homo sapiens*; pdb = Protein Data Bank (3D structures); c_int = *Ciona intestinalis*; b_flo = *Branchiostoma floridae*; s_pur = *Strongylocentrotus purpuratus*; s_kow = *Sa*ccoglossus kowalevskii; Pc = *Priapulus caudatus*; Dm = *Drosophila melanogaster*; La = *Lingula anatina*; Pa = *Phoronis australis*; c_tel = *Capitella teleta*; Ce = *Caenorhabditis elegans*; c_gig = *Crassostrea gigas*; a_cal = *Aplysia californica*; Xb = *Xenoturbella bockii*; n_vec = *Nem*atostella *vectensis*; e_dia = *Exaiptasia diaphana*; astro = *Astroides calycularis*; a_mil = *Acropora millep*ora; p_dam = *Pocillopora damicornis*; o_fav = *Orbicella faveolata*; a_aur = *Aurelia aurita*; m_vir = *Morbakka virulenta*; c_hem = *Clytia hemisphaerica*; h_vul = *Hydra vulgaris*.

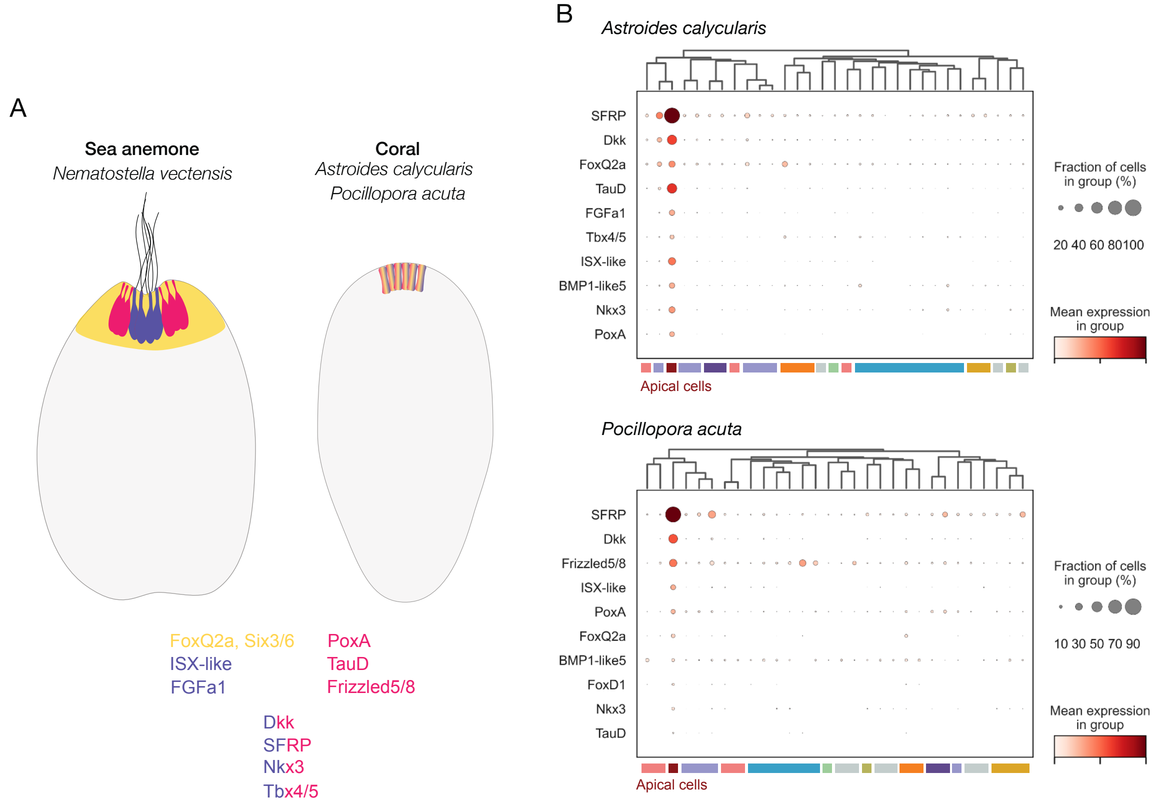

Fig. S14. Homology of coral planula apical cells to the apical organ of *Nematostella* planula. (A) Schematics illustrating spatial expression of known *Nematostella* apical organ genes. Gene names are color-coded according to their expression in specific areas/cell types. Yellow indicates the apical domain area; purple cells correspond to the apical tuft cells and magenta cells correspond to the surrounding larval neurons. Gene names colored half purple and half magenta indicate expression that is not specifically confined to either of the two apical organ cell types and is presumably present in both. References for spatial gene expression data: FoxQ2a, Six3/6 (*20*); FGFa1 (*6*, *20*, *31*); ISX-like, PoxA (*6*); TauD, Frizzled 5/8, SFRP (*33*); Dkk (*119*); Nkx3 (*30*, *120*); Tbx4/5 (*30*). (B) Dot plots showing expression of the *Nematostella* apical organ markers across *Astroides* (top) and *Pocillopora* (bottom) cell clusters. Gene names are listed on the left and cluster identities are indicated in colored panels at the bottom. The color coding of the clusters corresponds to the cell class annotations in Fig. 4. The color gradient indicates average expression, and the size of the dots indicates the percentage of cells expressing the gene.

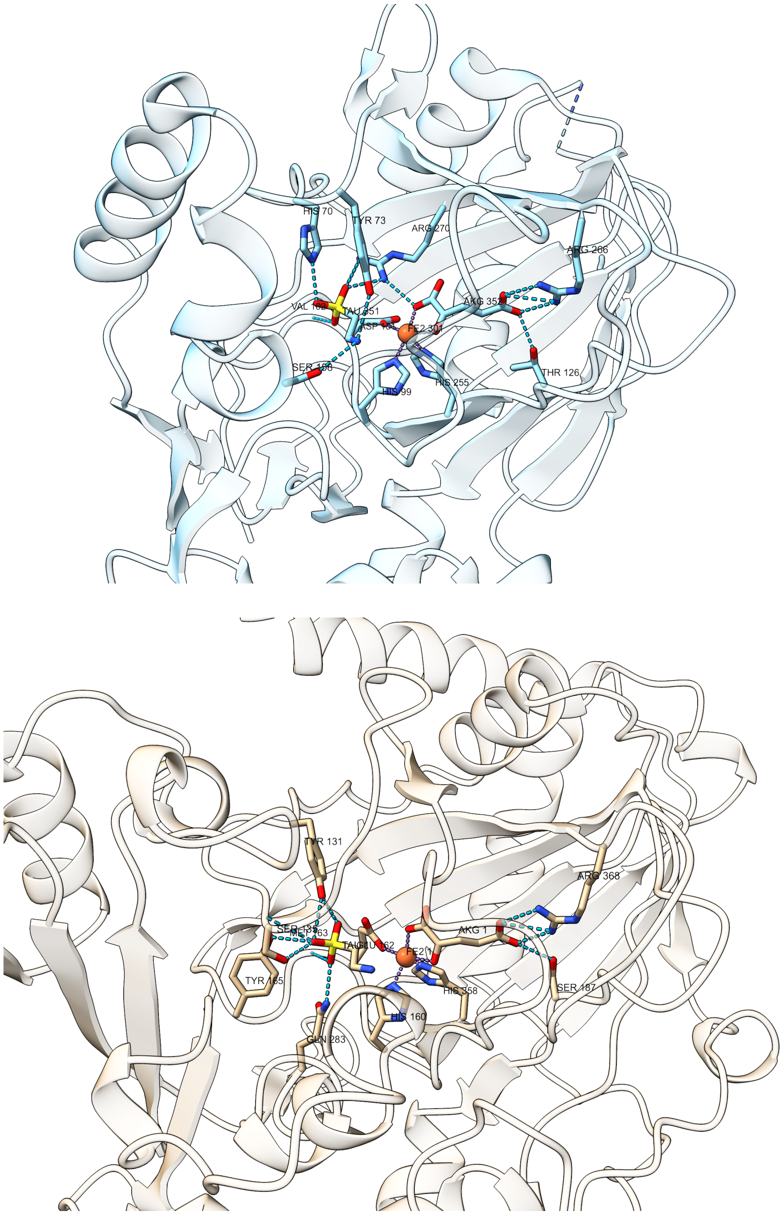

Fig. S15. Three-dimensional structure of the TauD protein from *E. coli* (top) and and the predicted structure from *Clytia* (bottom), showing bound ligands taurine (TAU), 2-oxoglutarate (alpha-ketoglutarate - AKG), and Fe^{2+} (FE2). The *E. coli* structure with ligands was determined by X-ray crystallography (pdb: 1GQW) (*68*). The *Clytia* structure was predicted from the protein sequence of the MSTRG.12764 gene and SMILES strings for ligands, using boltz-1 (*67*) - ligand positions were not constrained for the prediction. The protein chains were superimposed using the mmaker command from ChimeraX (*121*). Blue dashed lines indicate predicted hydrogen bonds and the purple dashed lines protein / metal ion coordination.

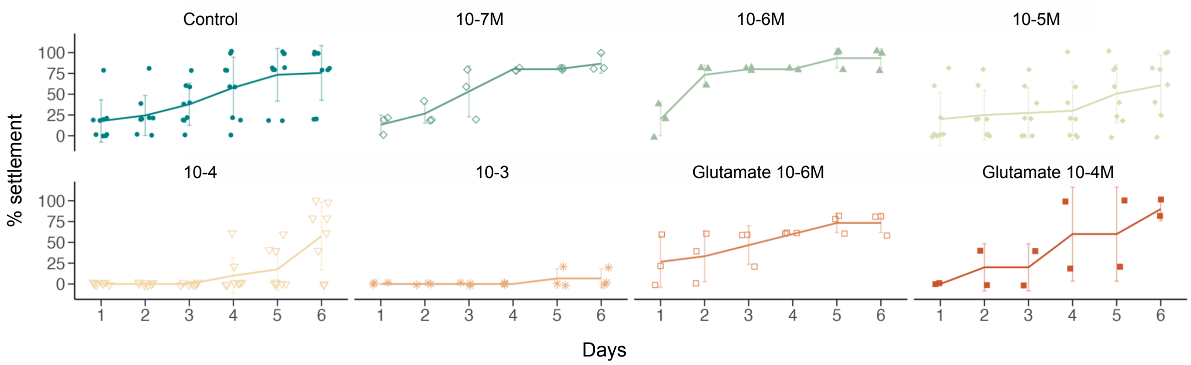

Fig. S16. Effect of Taurine and Glutamate treatment on *Astroides* settlement over time. Plots show the percentage of larvae that settled over the course of treatment (days) for each condition. Each dot represents the percentage of larvae that settled in an individual well, with 5 planulae used per treatment condition. Error bars indicate the standard deviation (see raw treatment data in table S4).

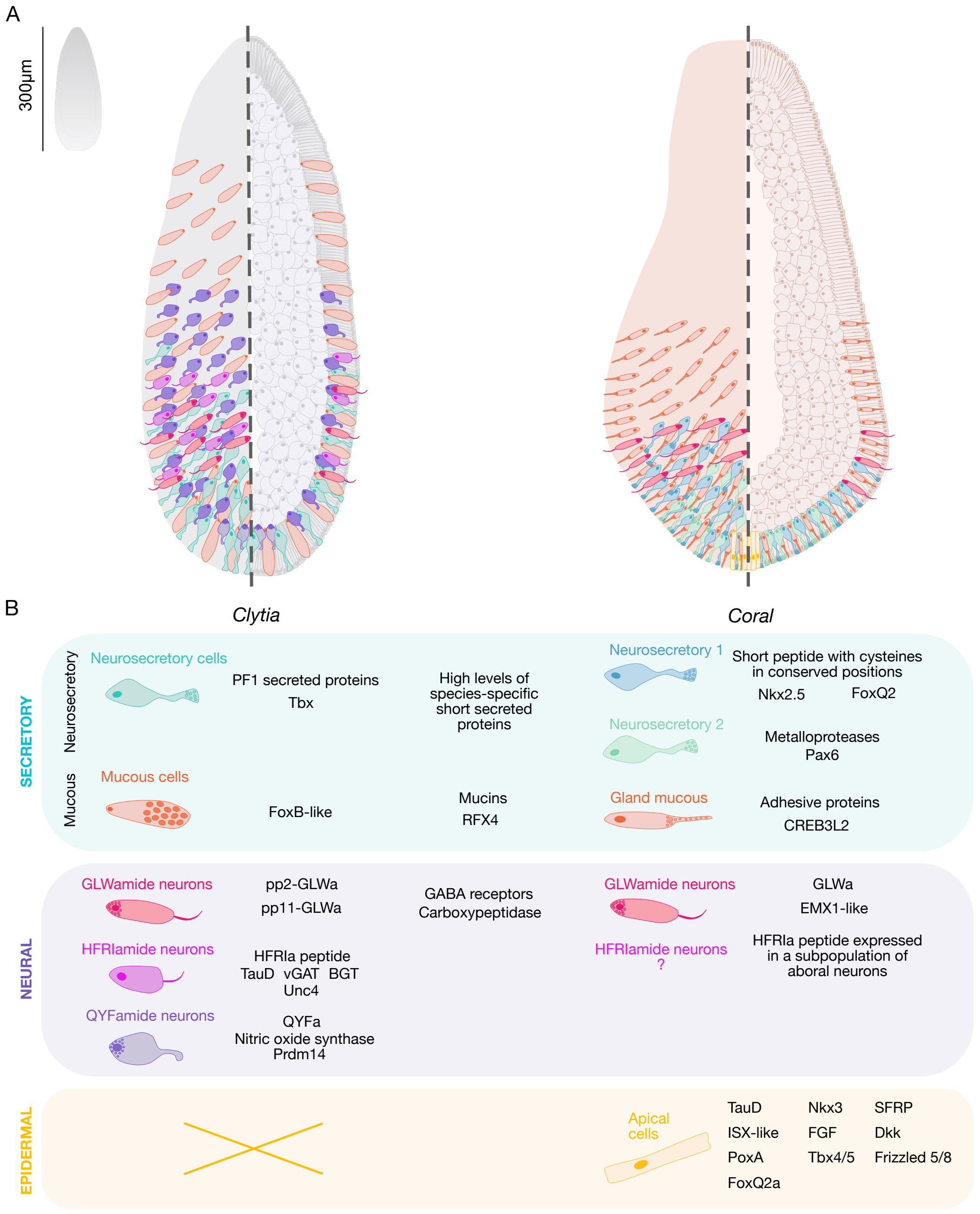

Fig. S17. Comparison of specialized aboral cell types between *Clytia* and coral planulae. (A) Schematics illustrating the distribution of aboral cell types in *Clytia* and coral planulae. The left section shows a surface view, and the right section corresponds to a medial view. The schematics of the two planulae are depicted at the same size for comparison purposes; however, the *Clytia* planula is substantially smaller than the *Astroides* planula. (B) Comparison of the molecular signatures of aboral cell types between *Clytia* and coral planulae.

Table S1. Aboral and oral enriched gene sets for *Clytia, Astroides* and *Pocillopora* planulae.

Table S2. Cluster marker gene sets for *Clytia*, *Astroides* and *Pocillopora* planula cell atlases.

Table S3. Genes shared between planula aboral cell types across species.

Table S4. Raw data from settlement assays.

Table S5. Primers and sequences used for ISH and HCR probe synthesis.

Table S6. Details of individual scRNA-seq libraries.

REFERENCES AND NOTES

1. W. A. Müller, A. Mitze, J.-P. Wickhorst, H. M. Meier-Menge, Polar morphogenesis in early hydroid development: Action of caesium, of neurotransmitters and of an intrinsic head activator on pattern formation. *Wilehm Roux. Arch. Dev. Biol.* **182**, 311–328 (1977).

2. M. B. Thomas, G. Freeman, V. J. Martin, The embryonic origin of neurosecretory cells and the role of nerve cells in metamorphosis in *Phialidium gregarium* (Cnidaria, Hydrozoa). *Int. J. Invertebr. Reprod. Dev.* **11**, 265–285 (1987).

3. S. Piraino, G. Zega, C. Di Benedetto, A. Leone, A. Dell'Anna, R. Pennati, D. C. Carnevali, V. Schmid, H. Reichert, Complex neural architecture in the diploblastic larva of Clava multicornis (Hydrozoa, Cnidaria). *J. Comp. Neurol.* **519**, 1931–1951 (2011).

4. N. Nakanishi, D. Yuan, D. K. Jacobs, V. Hartenstein, Early development, pattern, and reorganization of the planula nervous system in Aurelia (Cnidaria, Scyphozoa). *Dev. Genes Evol.* **218**, 511–524 (2008).

5. R. M. F. Attenborough, D. C. Hayward, U. Wiedemann, S. Forêt, D. J. Miller, E. E. Ball, Expression of the neuropeptides RFamide and LWamide during development of the coral Acropora millepora in relation to settlement and metamorphosis. *Dev. Biol.* **446**, 56–67 (2019).

6. E. Gilbert, C. Teeling, T. Lebedeva, S. Pedersen, N. Chrismas, G. Genikhovich, V. Modepalli, Molecular and cellular architecture of the larval sensory organ in the cnidarian Nematostella vectensis. *Development* **149**, dev200833 (2022).

7. K. Z. Sabin, S. Chen, E. M. Hill, K. J. Weaver, J. Yonke, M. Kirkman, W. B. Redwine, A. M. L. Klompen, X. Zhao, F. Guo, M. C. McKinney, J. L. Dewey, M. C. Gibson, Graded FGF activity patterns distinct cell types within the apical sensory organ of the sea anemone Nematostella vectensis. *Dev. Biol.* **510**, 50–65 (2024).

8. T. Leitz, M. Lay, Metamorphosin A is a neuropeptide. *Rouxs Arch. Dev. Biol.* **204**, 276–279 (1995).

9. J. Schmich, S. Trepel, T. Leitz, The role of GLWamides in metamorphosis of Hydractinia echinata. *Dev. Genes Evol.* **208**, 267–273 (1998).

10. T. Takahashi, N. Takeda, Insight into the molecular and functional diversity of cnidarian neuropeptides. *Int. J. Mol. Sci.* **16**, 2610–2625 (2015).

11. G. Krasovec, K. Pottin, M. Rosello, É. Quéinnec, J.-P. Chambon, Apoptosis and cell proliferation during metamorphosis of the planula larva of Clytia hemisphaerica (Hydrozoa, Cnidaria). *Dev. Dyn.* **250**, 1739–1758 (2021).

12. T. Cavalier-Smith, Origin of animal multicellularity: Precursors, causes, consequences—The choanoflagellate/sponge transition, neurogenesis and the Cambrian explosion. *Philos. Trans. R. Soc. Lond. B Biol. Sci.* **372**, 20150476 (2017).

13. D. Arendt, M. A. Tosches, H. Marlow, From nerve net to nerve ring, nerve cord and brain — Evolution of the nervous system. *Nat. Rev. Neurosci.* **17**, 61–72 (2016).

14. M. Lechable, A. Jan, A. Duchene, J. Uveira, B. Weissbourd, L. Gissat, S. Collet, L. Gilletta, S. Chevalier, L. Leclère, S. Peron, C. Barreau, R. Lasbleiz, E. Houliston, T. Momose, An improved whole life cycle culture protocol for the hydrozoan genetic model Clytia hemisphaerica. *Biol. Open* **9**, bio057034 (2020).

15. M. Gilis, A. Meibom, I. Domart-Coulon, O. Grauby, J. Stolarski, A. Baronnet, Biomineralization in newly settled recruits of the scleractinian coral Pocillopora damicornis. *J. Morphol.* **275**, 1349–1365 (2014).

16. A. Massé, I. Domart-Coulon, S. Golubic, D. Duché, A. Tribollet, Early skeletal colonization of the coral holobiont by the microboring Ulvophyceae Ostreobium sp. *Sci. Rep.* **8**, 2293 (2018).

17. C. Sinigaglia, H. Busengdal, L. Leclère, U. Technau, F. Rentzsch, The bilaterian head patterning gene six3/6 controls aboral domain development in a cnidarian. *PLOS Biol.* **11**, e1001488 (2013).

18. H. Marlow, M. A. Tosches, R. Tomer, P. R. Steinmetz, A. Lauri, T. Larsson, D. Arendt, Larval body patterning and apical organs are conserved in animal evolution. *BMC Biol.* **12**, 7 (2014).

19. K. A. Yankura, M. L. Martik, C. K. Jennings, V. F. Hinman, Uncoupling of complex regulatory patterning during evolution of larval development in echinoderms. *BMC Biol.* **8**, 143 (2010).

20. P. R. Steinmetz, R. Urbach, N. Posnien, J. Eriksson, R. P. Kostyuchenko, C. Brena, K. Guy, M. Akam, G. Bucher, D. Arendt, Six3 demarcates the anterior-most developing brain region in bilaterian animals. *Evodevo* **1**, 14 (2010).

21. S. Santagata, C. Resh, A. Hejnol, M. Q. Martindale, Y. J. Passamaneck, Development of the larval anterior neurogenic domains of Terebratalia transversa (Brachiopoda) provides insights into the diversification of larval apical organs and the spiralian nervous system. *Evodevo* **3**, 3 (2012).

22. V. S. Hunnekuhl, M. Akam, An anterior medial cell population with an apical-organ-like transcriptional profile that pioneers the central nervous system in the centipede Strigamia maritima. *Dev. Biol.* **396**, 136–149 (2014).

23. C. J. Lowe, M. Wu, A. Salic, L. Evans, E. Lander, N. Stange-Thomann, C. E. Gruber, J. Gerhart, M. Kirschner, Anteroposterior patterning in hemichordates and the origins of the chordate nervous system. *Cell* **113**, 853–865 (2003).

24. J. F. Ryan, M. E. Mazza, K. Pang, D. Q. Matus, A. D. Baxevanis, M. Q. Martindale, J. R. Finnerty, Pre-bilaterian origins of the Hox cluster and the Hox code: Evidence from the sea anemone, *Nematostella vectensis*. *PLOS ONE* **2**, e153 (2007).

25. R. Chiori, M. Jager, E. Denker, P. Wincker, C. Da Silva, H. Le Guyader, M. Manuel, E. Quéinnec, Are Hox genes ancestrally involved in axial patterning? Evidence from the hydrozoan Clytia hemisphaerica (Cnidaria). *PLOS ONE* **4**, e4231 (2009).

26. B. M. Steinworth, M. Q. Martindale, J. F. Ryan, Gene loss may have shaped the Cnidarian and Bilaterian Hox and ParaHox complement. *Genome Biol. Evol.* **15**, evac172 (2023).

27. E. Gilbert, J. Craggs, V. Modepalli, Gene regulatory network that shaped the evolution of larval apical organ in Cnidaria. *Mol. Biol. Evol.* **41**, msad285 (2024).

28. F. Rentzsch, J. H. Fritzenwanker, C. B. Scholz, U. Technau, FGF signalling controls formation of the apical sensory organ in the cnidarian Nematostella vectensis. *Development* **135**, 1761–1769 (2008).

29. J. Gerhart, C. Lowe, M. Kirschner, Hemichordates and the origin of chordates. *Curr. Opin. Genet. Dev.* **15**, 461–467 (2005).

30. C. Sinigaglia, H. Busengdal, A. Lerner, P. Oliveri, F. Rentzsch, Molecular characterization of the apical organ of the anthozoan Nematostella vectensis. *Dev. Biol.* **398**, 120–133 (2015).

31. J. Croce, L. Duloquin, G. Lhomond, D. R. McClay, C. Gache, Frizzled5/8 is required in secondary mesenchyme cells to initiate archenteron invagination during sea urchin development. *Development* **133**, 547–557 (2006).

32. S. Darras, J. Gerhart, M. Terasaki, M. Kirschner, C. J. Lowe, β-Catenin specifies the endomesoderm and defines the posterior organizer of the hemichordate Saccoglossus kowalevskii. *Development* **138**, 959–970 (2011).

33. A. M. Pani, E. E. Mullarkey, J. Aronowicz, S. Assimacopoulos, E. A. Grove, C. J. Lowe, Ancient deuterostome origins of vertebrate brain signalling centres. *Nature* **483**, 289–294 (2012).

34. A. Khadka, M. Martínez-Bartolomé, S. D. Burr, R. C. Range, A novel gene's role in an ancient mechanism: Secreted Frizzled-related protein 1 is a critical component in the anterior-posterior Wnt signaling network that governs the establishment of the anterior neuroectoderm in sea urchin embryos. *Evodevo* **9**, 1 (2018).

35. A. J. Poustka, A. Kühn, D. Groth, V. Weise, S. Yaguchi, R. D. Burke, R. Herwig, H. Lehrach, G. Panopoulou, A global view of gene expression in lithium and zinc treated sea urchin embryos: New components of gene regulatory networks. *Genome Biol.* **8**, R85 (2007).

36. P. Lapébie, A. Ruggiero, C. Barreau, S. Chevalier, P. Chang, P. Dru, E. Houliston, T. Momose, Differential responses to Wnt and PCP disruption predict expression and developmental function of conserved and novel genes in a cnidarian. *PLOS Genet.* **10**, e1004590 (2014).

37. J. Jumper, R. Evans, A. Pritzel, T. Green, M. Figurnov, O. Ronneberger, K. Tunyasuvunakool, R. Bates, A. Žídek, A. Potapenko, A. Bridgland, C. Meyer, S. A. A. Kohl, A. J. Ballard, A. Cowie, B. Romera-Paredes, S. Nikolov, R. Jain, J. Adler, T. Back, S. Petersen, D. Reiman, E. Clancy, M. Zielinski, M. Steinegger, M. Pacholska, T. Berghammer, S. Bodenstein, D. Silver, O. Vinyals, A. W. Senior, K.

Kavukcuoglu, P. Kohli, D. Hassabis, Highly accurate protein structure prediction with AlphaFold. *Nature* **596**, 583–589 (2021).

38. M. Mirdita, K. Schütze, Y. Moriwaki, L. Heo, S. Ovchinnikov, M. Steinegger, ColabFold: Making protein folding accessible to all. *Nat. Methods* **19**, 679–682 (2022).

39. M. van Kempen, S. S. Kim, C. Tumescheit, M. Mirdita, J. Lee, C. L. M. Gilchrist, J. Söding, M. Steinegger, Fast and accurate protein structure search with Foldseek. *Nat. Biotechnol.* **42**, 243–246 (2024).

40. L. Leclère, C. Horin, S. Chevalier, P. Lapébie, P. Dru, S. Peron, M. Jager, T. Condamine, K. Pottin, S. Romano, J. Steger, C. Sinigaglia, C. Barreau, G. Quiroga Artigas, A. Ruggiero, C. Fourrage, J. E. M. Kraus, J. Poulain, J.-M. Aury, P. Wincker, E. Quéinnec, U. Technau, M. Manuel, T. Momose, E. Houliston, R. R. Copley, The genome of the jellyfish Clytia hemisphaerica and the evolution of the cnidarian life-cycle. *Nat. Ecol. Evol.* **3**, 801–810 (2019).

41. S. Levy, V. Brekhman, A. Bakhman, A. Malik, A. Sebé-Pedrós, M. Kosloff, T. Lotan, Ectopic activation of $GABA_B$ receptors inhibits neurogenesis and metamorphosis in the cnidarian Nematostella vectensis. *Nat. Ecol. Evol.* **5**, 111–121 (2021).

42. M. S. Islam, T. M. Leissing, R. Chowdhury, R. J. Hopkinson, C. J. Schofield, 2-Oxoglutarate-dependent oxygenases. *Annu. Rev. Biochem.* **87**, 585–620 (2018).

43. H. Pasantes-Morales, Volume regulation in brain cells: cellular and molecular mechanisms. *Metab. Brain Dis.* **11**, 187–204 (1996).

44. P. Saransaari, S. S. Oja, Taurine and neural cell damage. *Amino Acids* **19**, 509–526 (2000).

45. J. Albrecht, A. Schousboe, Taurine interaction with neurotransmitter receptors in the CNS: An update. *Neurochem. Res.* **30**, 1615–1621 (2005).

46. A. Ferraioli, "Comparison of cell types across life cycle stages of the hydrozoan *Clytia hemisphaerica*," thesis, Sorbonne Université (2022).

47. I. Korsunsky, N. Millard, J. Fan, K. Slowikowski, F. Zhang, K. Wei, Y. Baglaenko, M. Brenner, P.-R. Loh, S. Raychaudhuri, Fast, sensitive and accurate integration of single-cell data with Harmony. *Nat. Methods* **16**, 1289–1296 (2019).

48. V. A. Traag, L. Waltman, N. J. van Eck, From Louvain to Leiden: Guaranteeing well-connected communities. *Sci. Rep.* **9**, 5233 (2019).

49. T. C. G. Bosch, Hydra and the evolution of stem cells. *Bioessays* **31**, 478–486 (2009).

50. S. Siebert, J. A. Farrell, J. F. Cazet, Y. Abeykoon, A. S. Primack, C. E. Schnitzler, C. E. Juliano, Stem cell differentiation trajectories in *Hydra* resolved at single-cell resolution. *Science* **365**, eaav9314 (2019).

51. N. Takeda, Y. Kon, G. Quiroga Artigas, P. Lapébie, C. Barreau, O. Koizumi, T. Kishimoto, K. Tachibana, E. Houliston, R. Deguchi, Identification of jellyfish neuropeptides that act directly as oocyte maturation-inducing hormones. *Development* **145**, dev156786 (2018).

52. T. Takahashi, M. Hatta, The importance of GLWamide neuropeptides in cnidarian development and physiology. *J. Amino Acids* **2011**, 424501 (2011).

53. E. Hayakawa, H. Watanabe, G. Menschaert, T. W. Holstein, G. Baggerman, L. Schoofs, A combined strategy of neuropeptide prediction and tandem mass spectrometry identifies evolutionarily conserved ancient neuropeptides in the sea anemone *Nematostella vectensis*. *PLOS ONE* **14**, e0215185 (2019).

54. T. Chari, B. Weissbourd, J. Gehring, A. Ferraioli, L. Leclère, M. Herl, F. Gao, S. Chevalier, R. R. Copley, E. Houliston, D. J. Anderson, L. Pachter, Whole-animal multiplexed single-cell RNA-seq reveals transcriptional shifts across Clytia medusa cell types. *Sci. Adv.* **7**, eabh1683 (2021).

55. Q. I. B. Lemaître, N. Bartsch, I. U. Kouzel, H. Busengdal, G. S. Richards, P. R. H. Steinmetz, F. Rentzsch, *NvPrdm14d*-expressing neural progenitor cells contribute to non-ectodermal neurogenesis in Nematostella vectensis. *Nat. Commun.* **14**, 4854 (2023).

56. S. Levy, A. Elek, X. Grau-Bové, S. Menéndez-Bravo, M. Iglesias, A. Tanay, T. Mass, A. Sebé-Pedrós, A stony coral cell atlas illuminates the molecular and cellular basis of coral symbiosis, calcification, and immunity. *Cell* **184**, 2973–2987.e18 (2021).

57. A. Sebé-Pedrós, B. Saudemont, E. Chomsky, F. Plessier, M.-P. Mailhé, J. Renno, Y. Loe-Mie, A. Lifshitz, Z. Mukamel, S. Schmutz, S. Novault, P. R. H. Steinmetz, F. Spitz, A. Tanay, H. Marlow, Cnidarian cell type diversity and regulation revealed by whole-organism single-cell RNA-Seq. *Cell* **173**, 1520–1534.e20 (2018).

58. J. Steger, A. G. Cole, A. Denner, T. Lebedeva, G. Genikhovich, A. Ries, R. Reischl, E. Taudes, M. Lassnig, U. Technau, Single-cell transcriptomics identifies conserved regulators of neuroglandular lineages. *Cell Rep.* **40**, 111370 (2022).

59. M. J. Layden, M. Boekhout, M. Q. Martindale, Nematostella vectensis achaete-scute homolog *NvashA* regulates embryonic ectodermal neurogenesis and represents an ancient component of the metazoan neural specification pathway. *Development* **139**, 1013–1022 (2012).

60. O. Castañeda, V. Sotolongo, A. M. Amor, R. Stöcklin, A. J. Anderson, A. L. Harvey, A. Engström, C. Wernstedt, E. Karlsson, Characterization of a potassium channel toxin from the Caribbean Sea anemone *Stichodactyla helianthus*. *Toxicon* **33**, 603–613 (1995).

61. Y. Moran, D. Praher, A. Schlesinger, A. Ayalon, Y. Tal, U. Technau, Analysis of soluble protein contents from the nematocysts of a model sea anemone sheds light on venom evolution. *Mar. Biotechnol.* **15**, 329–339 (2013).

62. Y. Y. Columbus-Shenkar, M. Y. Sachkova, J. Macrander, A. Fridrich, V. Modepalli, A. M. Reitzel, K. Sunagar, Y. Moran, Dynamics of venom composition across a complex life cycle. *eLife* **7**, e35014 (2018).

63. M. Y. Sachkova, M. Landau, J. M. Surm, J. Macrander, S. A. Singer, A. M. Reitzel, Y. Moran, Toxin-like neuropeptides in the sea anemone *Nematostella* unravel recruitment from the nervous system to venom. *Proc. Natl. Acad. Sci. U.S.A.* **117**, 27481–27492 (2020).

64. J. Wohlwend, G. Corso, S. Passaro, M. Reveiz, K. Leidal, W. Swiderski, T. Portnoi, I. Chinn, J. Silterra, T. Jaakkola, R. Barzilay, Boltz-1: Democratizing biomolecular interaction modeling. bioRxiv:624167 [Preprint] (2024). https://doi.org/10.1101/2024.11.19.624167.

65. J. M. Elkins, M. J. Ryle, I. J. Clifton, J. C. Dunning Hotopp, J. S. Lloyd, N. I. Burzlaff, J. E. Baldwin, R. P. Hausinger, P. L. Roach, X-ray crystal structure of *Escherichia coli* taurine/α-ketoglutarate dioxygenase complexed to ferrous iron and substrates. *Biochemistry* **41**, 5185–5192 (2002).

66. R. J. Huxtable, Physiological actions of taurine. *Physiol. Rev.* **72**, 101–163 (1992).

67. I. H. Lambert, D. M. Kristensen, J. B. Holm, O. H. Mortensen, Physiological role of taurine – From organism to organelle. *Acta Physiol.* **213**, 191–212 (2015).

68. P. A. V. Anderson, H. G. Trapido-Rosenthal, Physiological and chemical analysis of neurotransmitter candidates at a fast excitatory synapse in the jellyfish *Cyanea capillata* (Cnidaria, Scyphozoa). *Invert. Neurosci.* **9**, 167–173 (2009).

69. W. A. Müller, T. Leitz, Metamorphosis in the Cnidaria. *Can. J. Zool.* **80**, 1755–1771 (2002).

70. S. Berking, Taurine found to stabilize the larval state is released upon induction of metamorphosis in the hydrozon Hydractinia. *Rouxs Arch. Dev. Biol.* **197**, 321–327 (1988).

71. S. Berking, Is homarine a morphogen in the marine hydroid Hydractinia? *Rouxs Arch. Dev. Biol.* **195**, 33–38 (1986).

72. M. Walther, R. Ulrich, M. Kroiher, S. Berking, Metamorphosis and pattern formation in Hydractinia echinata, a colonial hydroid. *Int. J. Dev. Biol.* **40**, 313–322 (1996).

73. F. Bodo, J. Bouillon, Étude histologique du développement embryonnaire de quelques hydroméduses de Roscoff: *Phialidium hemisphaericum* (L.), Obelia sp. Péron et Lesueur, *Sarsia eximia* (Allman), *Podocoryne carnea* (Sars), *Gonionemus vertens* Agassiz. *Cah. Biol. Mar.* **9**, 69–104 (1968).

74. J. Vandermeulen, Studies on reef corals. II. Fine structure of planktonic planula larva of *Pocillopora damicornis*, with emphasis on the aboral epidermis. *Mar. Biol.* **27**, 239–249 (1974).

75. X. Li, S. Li, X. Huang, Y. Chen, J. Cheng, A. Zhan, Protein-mediated bioadhesion in marine organisms: A review. *Mar. Environ. Res.* **170**, 105409 (2021).

76. L.-S. He, G. Zhang, P.-Y. Qian, Characterization of two 20kDa-cement protein (cp20k) homologues in *Amphibalanus amphitrite*. *PLOS ONE* **8**, e64130 (2013).

77. M. Monier, V. Courtier-Orgogozo, Drosophila glue: A promising model for bioadhesion. *Insects* **13,** 734 (2022).

78. S. Savoca, D. Di Fresco, A. Alesci, G. Capillo, N. Spanò, Mucus secretions in Cnidarian, an ecological, adaptive and evolutive tool. *Adv. Oceanogr. Limnol.* **13**, 10.4081/aiol.2022.11054 (2022).

79. G. F. Ricardo, R. J. Jones, P. L. Clode, A. P. Negri, Mucous secretion and cilia beating defend developing coral larvae from suspended sediments. *PLOS ONE* **11**, e0162743 (2016).

80. B. E. Brown, J. C. Bythell, Perspectives on mucus secretion in reef corals. *Mar. Ecol. Prog. Ser.* **296**, 291–309 (2005).

81. H. Gröger, V. Schmid, Larval development in Cnidaria: A connection to Bilateria? *Genesis* **29**, 110–114 (2001).

82. S. Birch, D. Plachetzki, Multisensory integration by polymodal sensory neurons dictates larval settlement in a brainless cnidarian larva. *Mol. Ecol.* **32**, 3892–3907 (2023).

83. C. D. Bishop, B. P. Brandhorst, NO/cGMP signaling and HSP90 activity represses metamorphosis in the sea urchin *Lytechinus pictus*. *Biol. Bull.* **201**, 394–404 (2001).

84. S. Comes, A. Locascio, F. Silvestre, M. d'Ischia, G. L. Russo, E. Tosti, M. Branno, A. Palumbo, Regulatory roles of nitric oxide during larval development and metamorphosis in *Ciona intestinalis*. *Dev. Biol.* **306**, 772–784 (2007).

85. C. D. Bishop, A. Pires, S.-W. Norby, D. Boudko, L. L. Moroz, M. G. Hadfield, Analysis of nitric oxide-cyclic guanosine monophosphate signaling during metamorphosis of the nudibranch *Phestilla sibogae* Bergh (Gastropoda: Opisthobranchia). *Evol. Dev.* **10**, 288–299 (2008).

86. W. J. Biggers, A. Pires, J. A. Pechenik, E. Johns, P. Patel, T. Polson, J. Polson, Inhibitors of nitric oxide synthase induce larval settlement and metamorphosis of the polychaete annelid *Capitella teleta*. *Invertebr. Reprod. Dev.* **56**, 1–13 (2012).

87. Y. Zhang, L.-S. He, G. Zhang, Y. Xu, O.-O. Lee, K. Matsumura, P.-Y. Qian, The regulatory role of the NO/cGMP signal transduction cascade during larval attachment and metamorphosis of the barnacle *Balanus* (=*Amphibalanus*) *amphitrite*. *J. Exp. Biol.* **215**, 3813–3822 (2012).

88. N. Ueda, G. S. Richards, B. M. Degnan, A. Kranz, M. Adamska, R. P. Croll, S. M. Degnan, An ancient role for nitric oxide in regulating the animal pelagobenthic life cycle: Evidence from a marine sponge. *Sci. Rep.* **6**, 37546 (2016).

89. H. Song, O. H. Hewitt, S. M. Degnan, Arginine biosynthesis by a bacterial symbiont enables nitric oxide production and facilitates larval settlement in the marine-sponge host. *Curr. Biol.* **31**, 433–437.e3 (2021).

90. C. Carbonne, S. Comeau, P. T. W. Chan, K. Plichon, J.-P. Gattuso, N. Teixidó, Early life stages of a Mediterranean coral are vulnerable to ocean warming and acidification. *Biogeosciences* **19**, 4767–4777 (2022).

91. C. Kopp, I. Domart-Coulon, D. Barthelemy, A. Meibom, Nutritional input from dinoflagellate symbionts in reef-building corals is minimal during planula larval life stage. *Sci. Adv.* **2**, e1500681 (2016).

92. R. Cunning, R. A. Bay, P. Gillette, A. C. Baker, N. Traylor-Knowles, Comparative analysis of the *Pocillopora damicornis* genome highlights role of immune system in coral evolution. *Sci. Rep.* **8**, 16134 (2018).

93. A. Dobin, C. A. Davis, F. Schlesinger, J. Drenkow, C. Zaleski, S. Jha, P. Batut, M. Chaisson, T. R. Gingeras, STAR: Ultrafast universal RNA-seq aligner. *Bioinformatics* **29**, 15–21 (2013).

94. M. I. Love, W. Huber, S. Anders, Moderated estimation of fold change and dispersion for RNA-seq data with DESeq2. *Genome Biol.* **15**, 550 (2014).

95. H. García-Castro, N. J. Kenny, M. Iglesias, P. Álvarez-Campos, V. Mason, A. Elek, A. Schönauer, V. A. Sleight, J. Neiro, A. Aboobaker, J. Permanyer, M. Irimia, A. Sebé-Pedrós, J. Solana, ACME dissociation: A versatile cell fixation-dissociation method for single-cell transcriptomics. *Genome Biol.* **22**, 89 (2021).

96. B. Kaminow, D. Yunusov, A. Dobin, STARsolo: Accurate, fast and versatile mapping/quantification of single-cell and single-nucleus RNA-seq data. bioRxiv:442755 [Preprint] (2021). https://doi.org/10.1101/2021.05.05.442755.

97. F. A. Wolf, P. Angerer, F. J. Theis, SCANPY: Large-scale single-cell gene expression data analysis. *Genome Biol.* **19**, 15 (2018).

98. M. Kolmogorov, J. Yuan, Y. Lin, P. A. Pevzner, Assembly of long, error-prone reads using repeat graphs. *Nat. Biotechnol.* **37**, 540–546 (2019).

99. M. Pertea, G. M. Pertea, C. M. Antonescu, T.-C. Chang, J. T. Mendell, S. L. Salzberg, StringTie enables improved reconstruction of a transcriptome from RNA-seq reads. *Nat. Biotechnol.* **33**, 290–295 (2015).

100. B. J. Haas, A. Papanicolaou, M. Yassour, M. Grabherr, P. D. Blood, J. Bowden, M. B. Couger, D. Eccles, B. Li, M. Lieber, M. D. MacManes, M. Ott, J. Orvis, N. Pochet, F. Strozzi, N. Weeks, R. Westerman, T. William, C. N. Dewey, R. Henschel, R. D. LeDuc, N. Friedman, A. Regev, *De novo* transcript sequence reconstruction from RNA-seq using the Trinity platform for reference generation and analysis. *Nat. Protoc.* **8**, 1494–1512 (2013).

101. F. A. Simão, R. M. Waterhouse, P. Ioannidis, E. V. Kriventseva, E. M. Zdobnov, BUSCO: Assessing genome assembly and annotation completeness with single-copy orthologs. *Bioinformatics* **31**, 3210–3212 (2015).

102. W. R. Pearson, D. J. Lipman, Improved tools for biological sequence comparison. *Proc. Natl. Acad. Sci. U.S.A.* **85**, 2444–2448 (1988).

103. P. Kapli, T. Flouri, M. J. Telford, Systematic errors in phylogenetic trees. *Curr. Biol.* **31**, R59–R64 (2021).

104. R. D. Finn, P. Coggill, R. Y. Eberhardt, S. R. Eddy, J. Mistry, A. L. Mitchell, S. C. Potter, M. Punta, M. Qureshi, A. Sangrador-Vegas, G. A. Salazar, J. Tate, A. Bateman, The Pfam protein families database: Towards a more sustainable future. *Nucleic Acids Res.* **44**, D279–D285 (2016).

105. J. J. Almagro Armenteros, K. D. Tsirigos, C. K. Sønderby, T. N. Petersen, O. Winther, S. Brunak, G. von Heijne, H. Nielsen, SignalP 5.0 improves signal peptide predictions using deep neural networks. *Nat. Biotechnol.* **37**, 420–423 (2019).

106. C. Sinigaglia, D. Thiel, A. Hejnol, E. Houliston, L. Leclère, A safer, urea-based in situ hybridization method improves detection of gene expression in diverse animal species. *Dev. Biol.* **434**, 15–23 (2018).

107. S. Chevalier, A. Martin, L. Leclère, A. Amiel, E. Houliston, Polarised expression of FoxB and FoxQ2 genes during development of the hydrozoan *Clytia hemisphaerica*. *Dev. Genes Evol.* **216**, 709–720 (2006).

108. H. S. Bruce, G. Jerz, S. R. Kelly, J. McCarthy, A. Pomerantz, G. Senevirathne, A. Sherrard, D. A. Sun, C. Wolff, N. H. Patel, "Hybridization chain reaction (HCR) in situ protocol," *protocols.io* (2021); https://dx.doi.org/10.17504/protocols.io.bunznvf6.

109. O. Koizumi, S. Hamada, S. Minobe, K. Hamaguchi-Hamada, M. Kurumata-Shigeto, M. Nakamura, H. Namikawa, The nerve ring in cnidarians: its presence and structure in hydrozoan medusae. *Fortschr. Zool.* **118**, 79–88 (2015).

110. J. Schindelin, I. Arganda-Carreras, E. Frise, V. Kaynig, M. Longair, T. Pietzsch, S. Preibisch, C. Rueden, S. Saalfeld, B. Schmid, J.-Y. Tinevez, D. J. White, V. Hartenstein, K. Eliceiri, P. Tomancak, A. Cardona, Fiji: An open-source platform for biological-image analysis. *Nat. Methods* **9**, 676–682 (2012).

111. E. Kayal, B. Bentlage, M. Sabrina Pankey, A. H. Ohdera, M. Medina, D. C. Plachetzki, A. G. Collins, J. F. Ryan, Phylogenomics provides a robust topology of the major cnidarian lineages and insights on the origins of key organismal traits. *BMC Evol. Biol.* **18**, 68 (2018).

112. D. T. Schultz, S. H. D. Haddock, J. V. Bredeson, R. E. Green, O. Simakov, D. S. Rokhsar, Ancient gene linkages support ctenophores as sister to other animals. *Nature* **618**, 110–117 (2023).

113. E. Houliston, T. Momose, M. Manuel, Clytia hemisphaerica: A jellyfish cousin joins the laboratory. *Trends Genet.* **26**, 159–167 (2010).

114. M. A. Larkin, G. Blackshields, N. P. Brown, R. Chenna, P. A. McGettigan, H. McWilliam, F. Valentin, I. M. Wallace, A. Wilm, R. Lopez, J. D. Thompson, T. J. Gibson, D. G. Higgins, Clustal W and Clustal X version 2.0. *Bioinformatics* **23**, 2947–2948 (2007).

115. L. S. Babonis, C. Enjolras, J. F. Ryan, M. Q. Martindale, A novel regulatory gene promotes novel cell fate by suppressing ancestral fate in the sea anemone *Nematostella vectensis*. *Proc. Natl. Acad. Sci.* **119**, e2113701119 (2022).

116. E. Denker, E. Bapteste, H. Le Guyader, M. Manuel, N. Rabet, Horizontal gene transfer and the evolution of cnidarian stinging cells. *Curr. Biol.* **18**, R858–R859 (2008).

117. K. Sunagar, Y. Y. Columbus-Shenkar, A. Fridrich, N. Gutkovich, R. Aharoni, Y. Moran, Cell type-specific expression profiling unravels the development and evolution of stinging cells in sea anemone. *BMC Biol.* **16**, 108 (2018).

118. L. S. Babonis, M. Q. Martindale, *PaxA*, but not *PaxC*, is required for cnidocyte development in the sea anemone *Nematostella vectensis*. *Evodevo* **8**, 14 (2017).

119. P. N. Lee, K. Pang, D. Q. Matus, M. Q. Martindale, A WNT of things to come: Evolution of Wnt signaling and polarity in cnidarians. *Semin. Cell Dev. Biol.* **17**, 157–167 (2006).

120. H. Marlow, D. Q. Matus, M. Q. Martindale, Ectopic activation of the canonical wnt signaling pathway affects ectodermal patterning along the primary axis during larval development in the anthozoan Nematostella vectensis. *Dev. Biol.* **380**, 324–334 (2013).

121. E. C. Meng, T. D. Goddard, E. F. Pettersen, G. S. Couch, Z. J. Pearson, J. H. Morris, T. E. Ferrin, UCSF ChimeraX: Tools for structure building and analysis. *Protein Sci.* **32**, e4792 (2023).
